# Nocturnal selective pressures on the evolution of human musicality as a missing piece of the adaptationist puzzle

**DOI:** 10.3389/fpsyg.2023.1215481

**Published:** 2023-10-04

**Authors:** Marco Antonio Correa Varella

**Affiliations:** Department of Experimental Psychology, Institute of Psychology, University of São Paulo, São Paulo, Brazil

**Keywords:** music, song, night, esthetic behavior, evolutionary psychology, evolutionary musicology, chronotype, vocal communication

## Abstract

Human musicality exhibits the necessary hallmarks for biological adaptations. Evolutionary explanations focus on recurrent adaptive problems that human musicality possibly solved in ancestral environments, such as mate selection and competition, social bonding/cohesion and social grooming, perceptual and motor skill development, conflict reduction, safe time-passing, transgenerational communication, mood regulation and synchronization, and credible signaling of coalition and territorial/predator defense. Although not mutually exclusive, these different hypotheses are still not conceptually integrated nor clearly derived from independent principles. I propose *The Nocturnal Evolution of Human Musicality and Performativity Theory* in which the night-time is the missing piece of the adaptationist puzzle of human musicality and performing arts. The expansion of nocturnal activities throughout human evolution, which is tied to tree-to-ground sleep transition and habitual use of fire, might help (i) explain the evolution of musicality from independent principles, (ii) explain various seemingly unrelated music features and functions, and (iii) integrate many ancestral adaptive values proposed. The expansion into the nocturnal niche posed recurrent ancestral adaptive challenges/opportunities: lack of luminosity, regrouping to cook before sleep, imminent dangerousness, low temperatures, peak tiredness, and concealment of identity. These crucial night-time features might have selected evening-oriented individuals who were prone to acoustic communication, more alert and imaginative, gregarious, risk-taking and novelty-seeking, prone to anxiety modulation, hedonistic, promiscuous, and disinhibited. Those night-time selected dispositions may have converged and enhanced protomusicality into human musicality by facilitating it to assume many survival- and reproduction-enhancing roles (social cohesion and coordination, signaling of coalitions, territorial defense, antipredatorial defense, knowledge transference, safe passage of time, children lullabies, and sexual selection) that are correspondent to the co-occurring night-time adaptive challenges/opportunities. The nocturnal dynamic may help explain musical features (sound, loudness, repetitiveness, call and response, song, elaboration/virtuosity, and duetting/chorusing). Across vertebrates, acoustic communication mostly occurs in nocturnal species. The eveningness chronotype is common among musicians and composers. Adolescents, who are the most evening-oriented humans, enjoy more music. Contemporary tribal nocturnal activities around the campfire involve eating, singing/dancing, storytelling, and rituals. I discuss the nocturnal integration of musicality’s many roles and conclude that musicality is probably a multifunctional mental adaptation that evolved along with the night-time adaptive landscape.

## 1. Introduction

For the last 30 years, evolutionary psychology (EP) has advanced the comprehension of the evolved nature of human cognition (Schmitt and Pilcher, 2004; Tooby, 2020; Nettle and Scott-Phillips, 2023). However, its evolved temporal dynamicity (i.e., the daily and seasonal timings of cognitive functioning) has received little attention. The same happens in the literature on animal ornaments and primate vocalization (Hau et al., 2017; Piel, 2018). Most temporal concern in EP focuses on life-history development (Webster et al., 2014; Figueredo et al., 2021) or the menstrual cycle (Gildersleeve et al., 2014). Nevertheless, a comprehensive ‘Evolutionary Chronopsychology’ is needed, integrating EP and chronopsychology (Putilov, 2021), in which the seasonal, monthly, and circadian rhythms are also considered as evolved temporal signatures of mental adaptations integrated into the underlying chronobiological patterns of neural and endocrine systems. Early ethologists knew that “Behavior must occur not only in the right place and with the right orientation but also at the right moment. Quite generally, releasing mechanisms, whether innate or acquired, are programmed so as to set off adequate behavior at the teleonomically correct time” (Lorenz, 1981, p. 231).

There is heuristic value in considering the evolved temporal dimension of cognition. Jonason et al. (2013) predicted and found that individuals with high dark-triad personality (Machiavellianism, psychopathy, and narcissism) are evening-oriented because the nocturnal context favors the expression of such self-centered/antisocial tendencies. Their *cognitive niche hypothesis* has been corroborated (Akram et al., 2019; Porfírio and Varella, 2022). Similarly, Varella et al. (2021) predicted that evening-oriented individuals would be less compliant with the COVID-19 pandemic safety guidelines based on evolutionary consideration of some recurrent nocturnal challenges/opportunities (dangerousness, peak tiredness, and concealment of identity) and how it might have amplified risk-taking and rule-breaking tendencies in evening-oriented individuals. Their *eveningness epidemiological liability hypothesis* has been corroborated (Li, 2022). In general, phenotypic correlations between psychological traits show significant and substantial genetic mediation and the individual variation of psychological traits that have been studied shows significant and substantial genetic influence (Polderman et al., 2015; Plomin et al., 2016); thus, the genetic basis necessary for those evolutionary propositions is plausible.

Here, I apply the evolutionary perspective to the temporal dimension of human musicality, connecting evolutionary musicology (Wallin, 1991; Brown et al., 2000; Fitch, 2015) with evolutionary chronopsychology. The heuristic value of this endeavor might help advance and integrate the evolutionary debate concerning musicality. First, I outline definitions and the confluence of evidence indicating the evolved and multifunctional nature of human musicality. Then, I present the ancestral adaptive explanations for musicality stressing that they are not mutually exclusive, conceptually integrated, or clearly derived from independent principles. I fill this gap by proposing that the night-time selective landscape is the missing piece of the adaptationist puzzle of human musicality.

I put forward the *Nocturnal Evolution of Human Musicality and Performativity Theory* (NEHMPT). I provide evidence for the evolved expansion of human nocturnal activities and argue that the recurrent night-time ancestral adaptive challenges/opportunities (i.e., *lack of luminosity, low temperatures, regroup to cook before sleep, dangerousness, peak tiredness, and easy concealment of identity*) might have molded human musicality from ape-like protomusicality, in relation to the concomitant nocturnal ecosocial contexts. I present and discuss the nocturnal signatures of musicality. I hope to (i) explain why humans are more musical than other great apes, (ii) explain the evolution of musicality from independent principles, (iii) explain and interrelate the various seemingly unconnected features/functions of musicality, and (iv) integrate different ancestral adaptive values proposed (knowledge transference, social cohesion, signaling of coalitions, territorial/antipredatorial defense, safe passage of time, children lullaby, and sexual selection). I expand on previous attempts to relate the night-time with the evolution of musicality (Jordania, 2011; Putilov, 2014; Nowell, 2018; Varella and Valentova, 2023) and on previous attempts to synthesize existing adaptive hypotheses into a comprehensive evolutionary framework for human musicality (Savage P. et al., 2021).

### 1.1. Evolved propensities to develop a multifaceted musical cognition

Musicality has many meanings, such as a potential/propensity/predisposition, a universal capacity/ability, or an individual difference aptitude/talent for music engagement (Hallam, 2006). It can be understood as the species-specific propensity (i.e., genotypic or “potential” musicality) to ontogenetically develop an integrated set of broad musically related psychological capacities (phenotypic or “operational” musicality), including general capacities (e.g., auditory scene analysis, creativity, play, emotional recognition, and language), and the narrow musically specific melodic/harmonic and rhythmic cognition, subdivided into cognitive capacities for musical production (creation and imitation), musical discrimination, appreciation and memorization, and musical motivations (*cf.* Peretz and Coltheart, 2003; Jackendoff and Lerdahl, 2006; Bispham, 2009; Honing et al., 2015a; Leongómez et al., 2022). Music, as the sociocultural product of musical behavior and evocated/enabled by musical capacities/propensities, can be defined as mostly acoustic communication that intentionally uses variations in pitch, contour, rhythm, tempo, timbre, and volume in a combined and organized way according to an internal generative grammar forming harmonized and rhythmic melodies exploring repetition, modifications and expectation through the use of voice, body, and/or instruments leading to intense and shared esthetic and emotional reactions (*cf.* Jackendoff and Lerdahl, 2006; Peretz, 2006; Mithen, 2009; Honing et al., 2015a; Mehr et al., 2021; Savage P. E. et al., 2021). Music is a costly activity of sound-focused elaboration that produces an extraordinary and esthetically crafted sonic temporal sequence and engaging performance that attracts attention and inspires engagement and esthetic evaluations aligned with emotional and interpersonal reactions (Dissanayake, 2008; Varella et al., 2011, 2012, 2017). Beyond sound, other multimodal factors comprise musical activities, such as the visual self-presentation and movements of those musically engaged, including dancing, and the related linguistic and socio-historic aspects of music (Miller, 2000; Cross and Morley, 2002, 2008; Dissanayake, 2008; Fink et al., 2021).

**Figure 1:**
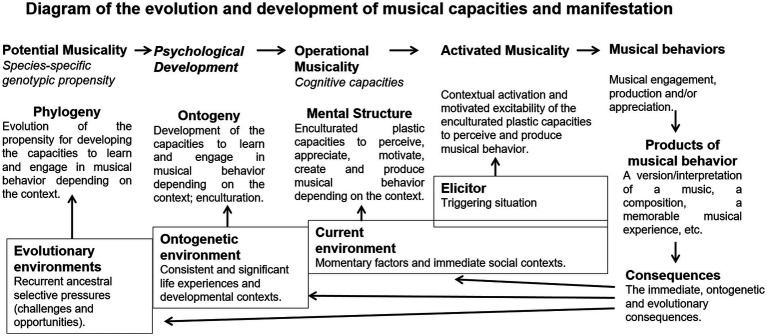
Diagram of the evolution and development of musical capacities and manifestation.

Figure 1 organizes the evolution and development and represents the relationship between musical propensity, capacity, behavior, and product.

Evolutionary musicology approaches human musicality phylogenetically and (non)adaptively (Wallin, 1991; Brown et al., 2000; Fitch, 2015). For evolutionary psychological purposes, the focus is on the operational cognitive traits and their corresponding genotypic propensity, not so much on manifested behavior or its products (Honing and Ploeger, 2012; Varella et al., 2013, 2017). Some authors consider musical behavior as a recent by-product of mostly preexisting general (not music-specific) cognitive capacities that evolved because of other-than-musical reasons (Spencer, 1875; Pinker, 1997; Patel, 2018). In fact, musical activities recruit many general capacities (e.g., attention, creativity), but there are also many music specificities (Peretz and Coltheart, 2003; Samson et al., 2009; Zatorre, 2015) that require their own conjoint evolutionary explanations.

Moreover, the individual variation in human musicality *per se* has a considerable genetic component: between 44 and 90% of the individual variation on musical ability is inherited (Coon and Carey, 1989), between 71 and 80% of the individual variation on pitch discrimination is inherited (Drayna et al., 2001), between 38 and 51% of the individual variation on musical aptitude, and between 9 and 57% of music achievement is inherited (Mosing et al., 2015). As for musical ability, the individual variance in musical practice also is substantially heritable (40–70%), and the association between both practice and ability is predominantly genetic (Mosing et al., 2014). Furthermore, a genome-wide association study has identified 69 genetic variants that influence variation in self-reported beat synchronization ability (Niarchou et al., 2022). Some musically related genetic variations are in chromosomes 4, 8, 12, and 17 (Tan et al., 2014). Importantly, the polygenic score for rhythmicity predicts melodic capacities and music-related outcomes but does not predict traits that are not directly related to music (Wesseldijk et al., 2022), suggesting that there is specificity and inherited individual variation which are necessary for there is selection to operate (Croston et al., 2015).

Furthermore, a nomological network of converging empirical evidence (Schmitt and Pilcher, 2004) has been assembled strongly indicating that human musicality exhibits the necessary hallmarks for biological adaptations (see Table 1). Human musicality has homologous precursors, adaptive convergence with songs of other species, antiquity, universality (across history, cultures, and individuals), heritability, precocity, standard ontogeny, and intrinsic motivation; it conveys pleasure and emotion, and exhibits complex cognitive modularity and neurological organization; it is beneficial to health, sexual, and psychological domains; and it is associated with social roles and cultural importance (Miller, 2000; Huron, 2001; Cross and Morley, 2002, 2008; Peretz, 2006; Dissanayake, 2008; Varella et al., 2010; Honing and Ploeger, 2012; Mehr et al., 2021). Overall, the full account of human musicality requires consideration of primordial acoustic/sonic capacities (precursors), ancient music-specific secondary adaptations, and recent cooptation of non-musical capacities within cultural dynamics (Varella et al., 2012; Trainor, 2021).

**Table 1:** Compilation of the 15 types of cross-disciplinary pieces of evidence pointing to the probable evolved status of music propensities and capacities.

Miller (2000)	Huron (2001)	Cross and Morley (2002)	Peretz (2006)	Dissanayake (2008)	Varella et al. (2010)	Honing and Ploeger (2012)	Mehr et al. (2021)
Universality across cultures	Universality across cultures	Universality across cultures	Universality across cultures	Universality across cultures	Universality across cultures	Universality across cultures	Universality across cultures
	Roles and cultural importance	Roles and cultural importance	Roles and cultural importance	Cultural importance	Cultural importance	Roles and cultural importance	
Spontaneous and precocious development		Spontaneous and precocious development	Spontaneous and precocious development	Spontaneous and precocious development	Spontaneous and precocious development	Spontaneous and precocious development	Precocious development
Neural specialization	Neural specialization	Neural specialization	Neural specialization		Neural specialization	Neural specialization	Neural specialization
Antiquity	Antiquity	Antiquity	Antiquity		Antiquity	Antiquity	Antiquity
Adaptive convergence	Adaptive convergence	Adaptive convergence		Adaptive convergence	Adaptive convergence	Adaptive convergence	Adaptive convergence
Plausible adaptive value(s)	Plausible adaptive value(s)		Plausible adaptive value(s)	Plausible adaptive value(s)	Plausible adaptive value(s)	Plausible adaptive value(s)	Plausible adaptive value(s)
Cognitive modularity	Cognitive modularity		Cognitive modularity		Cognitive modularity	Cognitive modularity	
Heritability and Genetic specificity	Genetic specificity		Heritability and Genetic specificity		Heritability and Genetic specificity	Genetic specificity	
Costliness	Costliness			Costliness	Costliness		
Universality across individuals		Universality across individuals	Universality across individuals		Universality across individuals		
			Highly positive and rewarding	Highly positive and rewarding	Highly positive and rewarding		
Evoke strong emotions			Evoke strong emotions		Evoke strong emotions		
		Homology of precursors	Homology of precursors				
Complex design							Complex design

### 1.2. Ancestral adaptive values of human musicality

The inquiry about the evolution of musicality is legitimate and remains open to debate and investigation (Kalinowski et al., 2021; Mehr et al., 2021; Savage P. et al., 2021; Savage P. E. et al., 2021). Since Darwin and Spencer initially discussed it (Kleinman, 2015), evolutionary theorizations about musicality have improved and diversified. Many empirical articles (e.g., Hagen and Bryant, 2003; Varella et al., 2010, 2022; Pearce et al., 2016), review articles (e.g., Hauser and McDermott, 2003; Fitch, 2006; Snowdon et al., 2015; Killin, 2018; Fink et al., 2021), books (e.g., Wallin et al., 2000; Mithen, 2005; Ball, 2010; Morley, 2013; Honing, 2018), and special issues (Peretz, 2006; Honing et al., 2015b; Nikolsky and Perlovsky, 2020) have been published, including guiding principles to improve/advance the field (Honing and Ploeger, 2012; Fitch, 2015; Merker et al., 2015; Currie and Killin, 2016).

The proposed evolutionary hypotheses are focused on different recurrent adaptive problems that human (proto)musicality possibly solved on ancestral environments: *mate attraction/bonding and intrasexual competition* (e.g., Darwin, 1871; Zahavi and Zahavi, 1999; Miller, 2000; Varella et al., 2017, 2022), *group cohesion and social bonding* (e.g., Brown, 2000; Dissanayake, 2008; Launay et al., 2016; Savage P. et al., 2021), *mother–infant bonding* (e.g., Trehub, 2003; Dissanayake, 2008; Mehr et al., 2021; Savage P. et al., 2021), *mood/emotional induction/regulation and synchronization* (Eibl-Eibesfeldt, 1989; Snowdon et al., 2015), *coalitional signaling* (Hagen and Bryant, 2003; Mehr et al., 2021), *territorial/predator defense* (Hagen and Hammerstein, 2009; Jordania, 2011; Hagen, 2022), *group effort, perceptual and motor skill development, safe time-passing, transgenerational communication* (Huron, 2001), *conflict reduction, and social tolerance* (Huron, 2001; Fukui and Toyoshima, 2023). Twenty years ago, there was a ‘lack of empirical evidence’ and ‘little basis’ on which to distinguish among the various evolutionary hypotheses about human musicality (Hauser and McDermott, 2003). Despite the empirical advances (e.g., Loersch and Arbuckle, 2013; Marin and Rathgeber, 2022), it is still ‘premature’ and ‘unjustified’ to rule out alternative hypotheses (Bowling et al., 2021). More empirical testing is required, and there is a still need for theoretical development.

### 1.3. The issue of integrating the evolutionary explanations for musicality and its functions

Different accounts for the evolution of musicality rely on one or a few major factors influencing the hominin evolution (i.e., since the *Pan* and human lineage separated 6–8 Ma [*Megaanni*; million years] ago, Langergraber et al., 2012). Miller (2000) stressed the increase in brain size and monogamy; Merker (1999, 2021), the necessity of attracting exogamously migrating females; Dissanayake (2008), Mehr et al. (2021) and Leongómez et al. (2022), the increase in immaturity and dependence of human offspring; Launay et al. (2016), Fink et al. (2021), and Savage P. et al. (2021), the increase in group size and a ‘bonding gap’ in human sociality; Jordania (2011) and Hagen (2022) emphasized the increase in predation risk and intergroup conflict; and Benítez-Burraco and Nikolsky (2023) focused on self-domestication. Because those factors were recurrent and ancient enough during hominin evolution, their effects are likely complementary. This makes most of the evolutionary explanations for human musicality non-exclusive and mutually compatible (Menninghaus, 2019; De Tiège et al., 2021).

As human musicality is closely related to acoustic communications in other animals (Miller, 2000; Hauser and McDermott, 2003; Fitch, 2006; Snowdon et al., 2015; Honing, 2018), it might also serve the adaptive functions found in other animals’ signals, i.e., courtship displays and mate-bonding, ritualized combat, territorial defense/advertisement, coalitional displays, social bonding, and group-cohesion signaling (De Tiège et al., 2021). Furthermore, human musicality plays many important roles not restricted to signaling, indicating it might have accumulated concomitant adaptive values. The functioning of human musicality delivers (1) health, (2) psychological, (3) sexual, and (4) social benefits: (1) Music listening alleviates pain, diminishes stress and anxiety, reduces blood glucose, boosts immunity, and increases the quality of post-operatory, pregnancy, childbirth, and the health of the newborn baby (North and Hargreaves, 2008; Finn and Fancourt, 2018; McCaffrey et al., 2020); (2) listening to music makes people more positive, more alert, and focused on the present (Sloboda et al., 2001), and it also counteracts insomnia, improves sleep onset, latency, and quality (Feng et al., 2018). Despite that individuals who self-select musical training tend to have better cognitive and academic performance at baseline, musical training has a small but positive impact on cognitive skills and academic achievement (Román-Caballero et al., 2022). Musical training seems to enhance verbal intelligence and executive function (Schellenberg, 2004, 2011; Moreno et al., 2011), phonological awareness (Gordon et al., 2015), verbal working and long-term memories (Ho et al., 2003; Franklin et al., 2008), language development, literacy, numeracy, intelligence, general attainment, creativity, fine motor coordination, concentration, self-confidence, emotional sensitivity, social skills, teamwork, self-discipline, and relaxation (Hallam, 2010); (3) listening to music makes women find men more attractive and increases men’s and women’s desire to date other-sex target individuals (May and Hamilton, 1980; Marin et al., 2017; Chang et al., 2021; Marin and Rathgeber, 2022). The ability to sing, play a musical instrument, or dance is considered attractive in a mate (Weege et al., 2012; Charlton, 2014; Wade et al., 2015; Röder et al., 2016; Bongard et al., 2019; Valentova et al., 2019). Dancing with someone is perceived as an effective flirting tactic (Wade et al., 2021; Kennair et al., 2022). Music is sometimes present in individuals’ sexual fantasies (Lehmiller, 2018), it enhances feelings of sexual arousal when paired with a consensual sex story (Wan and Lalumière, 2017), and it has been effectively used in ‘sex playlists’ during sexual activities (Van Bohemen et al., 2018) and in couple/sexual therapies (Duba and Roseman, 2012; Micoogullari et al., 2021); (4) music functions as a way to express emotions, get esthetic enjoyment, entertain, communicate, represent symbolically, manage self-identity/self-awareness/mood/interpersonal relationships, coordinate action, enforce social norms, validate social institutions and rituals, contribute to continuity/stability of culture, and contribute to integration of society (Merriam, 1964; Hargreaves and North, 1999; Schäfer et al., 2013; Clayton, 2016). Thus, far from being useless, musicality offers many evolutionarily relevant benefits.

The different evolutionary hypotheses for musicality and its multifunctional nature are still not conceptually integrated. De Tiège et al. (2021) offer an animal signaling theory as a unifying framework for the multifunctional communicative nature of human musicality and artisticality (*cf.* Zahavi and Zahavi, 1999; Miller, 2000; Varella et al., 2011; Fink et al., 2021). More restricted to musicality and to mother–offspring and intergroup interactions, Mehr et al. (2021) have also employed signaling theory to propose an account that human musicality evolved as a credible signaling device indicating parental attention and coalitional quality. Savage P. et al. (2021) proposed an overarching social bonding function of musicality connecting mother–offspring bonding, romantic pair bonding, and in-group bonding. Others have explained the multiple functions of musicality as a fortuitous sequence of co-optations/exaptations to perform new functions followed by new selection (e.g., Trainor, 2015; Garfinkel, 2018; Dubourg et al., 2021).

I integrate these models by arguing that human musicality is multifunctional because the need and situation for accomplishing most of those functions were recurrently co-occurring during night-time.

### 1.4. The issue of deriving the design features and functions of musicality from independent principles

The explanations for the design features and functions of musicality should rely on processes/regularities external to music cognition *per se*. The musical capacities and functions should be derived from independent principles (Pinker, 2005), the same way the designs and functions of the human eye and the human cochlea correspondent to the optics and acoustic principles, respectively (*cf.* Pinker, 1997, 2005; Trainor, 2015).

Pinker (2005) argued that the proposed adaptive functions for musicality (e.g., group cohesion) are circular and consequently not proper explanations because they do not rely on a set of principles independent of the part of our psychology being explained. According to him, such an explanation only begs the question of why harmonically and rhythmically organized sound sequences should in principle foster group cohesion. “Generating and sensing sequences of sounds is not an independently motivated solution to the problem of maintaining group solidarity, in the way that, say, the emotion of empathy, or a motive to punish free riders, is part of such a solution” (p. xv).

Here, I suggest that the night-time features (i.e., lack of luminosity, low temperatures, regrouping to cook before sleep, dangerousness, peak tiredness, and concealment of identity) are the independent principles necessary to a new comprehensive evolutionary understanding of human musicality.

### 1.5. Tree-to-ground sleep transition, fire, and the expansion toward the nocturnal niche

The common ancestor of all primates was likely nocturnal, and apes are considered as cathemeral (i.e., activity not restricted to a particular time of day or night) (Wu et al., 2017). Male Orangutans in captivity perform premeditated nocturnal long calls (Samson et al., 2014). Wild Chimpanzees exhibit episodes of rain-dancing, pant-hoot chorusing, or “carnival displays” that can last from hours to the whole night (Dufour et al., 2015; Merker et al., 2015). During night-time, Chimpanzees can play (Tagg et al., 2018), feed, vocalize, travel, and mate (Anderson et al., 2019), taking 1.8% of their awake activity (Tagg et al., 2018). Interestingly, at night-time, Chimpanzees perform twice as many long-distance vocalizations as social proximity calls (Pruetz, 2018). Every night, and at a rate of almost twice an hour, Chimpanzees perform such long-distance communication maintaining contact with those traveling nearby (Zamma, 2014; Pruetz, 2018). This indicates a tendency for loud nocturnal vocalizations in Chimpanzees.

Importantly, there is a spontaneous inclination toward some level of rhythmic synchronization and entrainment in chimpanzees (Hattori et al., 2013) and bonobos (Large and Gray, 2015). Spontaneous drumming is also found in Chimpanzees (Arcadi et al., 1998; Dufour et al., 2015). Finally, Chimpanzees prefer consonant music (Sugimoto et al., 2009) and African and Indian traditional music over silence (Mingle et al., 2014); human instrumental music seems to increase affiliative behavior in captive Chimpanzees, while vocal music seems to decrease their agonistic behavior (Videan et al., 2007). Thus, the common ancestor between the pan and the hominin lineage (6–8 Ma ago, Langergraber et al., 2012) likely exhibited a tendency toward nocturnal vocalizations in this much older basic protomusicality, which was later ‘upgraded’ in hominin ancestors during Pleistocene.

Primate lineages rapidly adapt their circadian pattern to diurnal or nocturnal niches (Ankel-Simons and Rasmussen, 2008). During hot days and maize season, Chimpanzees expand their nocturnal activities after twilight feeding from the unguarded crops (Lacroux et al., 2022). This general ape-like flexible and occasional evening-oriented behavior could have enabled ancestral hominins’ adaptability (Tagg et al., 2018). Furthermore, ancient humans were selected for the nocturnal niche beyond the basic ape-like evening orientation (Coolidge and Wynn, 2006).

Humans have a uniquely evolved sleep pattern, suggesting an ancestral selective pressure to rapidly accomplish sleep necessities (Nunn and Samson, 2018). Human sleep is shorter, deeper, and without much movement and exhibits more occasions of REM sleep making it more efficient (improving memory consolidation and insightful problem solving while dreaming) and freeing the evening for socialization (Coolidge and Wynn, 2006; Samson and Nunn, 2015; Nunn et al., 2016; Nunn and Samson, 2018). Based on variation across primates, humans should sleep 9.55 h but actually sleep 7 h on average worldwide, making us a phylogenetic outlier (Nunn and Samson, 2018). Given that in Central Africa, the sun sets approximately at 18:00 and rises at 6:00, humans waking up at sunrise, even without a noon nap, could have had some 5 h awake after sunset (30% of the awake time). The tree-to-ground sleep transition (Coolidge and Wynn, 2006; Samson and Nunn, 2015; Fruth et al., 2018), predation risk (Samson, 2021), and use of fire (Coolidge and Wynn, 2006; Samson and Nunn, 2015; Fruth et al., 2018; Killin, 2018) represent significant evolutionary factors concerning the expansion of human evening activities setting the stage for unique subsequent evolutionary changes.

Early *Homo erectus* might have opportunistically and sporadically made use of fire as early as 1.6 Ma ago (Roebroeks and Villa, 2011; Gowlett, 2016). This is concomitant to the increased out-of-Africa migration toward colder locations (Gowlett, 2006; Brown et al., 2009), the appearance of the more developed Acheulean technology used in the butchery of fauna (Diez-Martín et al., 2015), and to the origin of digestive adaptations for cooked food, such as gracile teeth and jaw (Wrangham, 2017). During the early opportunistic phase of fire use, ancestral hominins were adapting to the progressively dry and fire-prone environments (Parker et al., 2016) and were already cooking fish 780 ka (i.e., *kiloanni*; thousand years) ago (Zohar et al., 2022). Wildfires tend to scare, confuse, and kill prey and predators, opening predator-free scavenging/hunting grounds (Herzog et al., 2020).

Despite the ongoing debate, this initial timeline of fire use is also considered to be aligned with the tree-to-ground nesting transition by the fully bipedal early *Homo erectus* (Coolidge and Wynn, 2006; Wrangham, 2009; Samson and Nunn, 2015; Fruth et al., 2018). Nesting on the ground eliminates the danger of eventually falling out of the tree; the ground makes for a more stable nest enabling a more uninterrupted and efficient sleep, so much so that nesting on the ground or in lower branches happens sometimes in Chimpanzees when there is less predation risk (Hernandez-Aguilar et al., 2013; Fruth et al., 2018; Pozzi et al., 2022). Thus, sleeping hidden in caves, probably protected by sentinels (Samson and Nunn, 2015; Samson et al., 2017) capable of stone throwing (which appeared 2 Ma ago, Lombardo and Deaner, 2018) and by sporadic use of fire arguably, could have favored the tree-to-ground nesting in *Homo erectus* speeding sleep efficiency freeing nocturnal awake time. The initial phase of sporadic use of fire was followed by a transitory phase that culminated more than a million years later in habitual fire use (Gowlett, 2016), dating 400–300 ka ago in Neanderthals and slightly later in early *Homo sapiens* (Rolland, 2004; Roebroeks and Villa, 2011; Scott and Hosfield, 2021). So far, the earliest fossil of *Homo sapiens* dates 315 ka (Hublin et al., 2017), this period is considered to be the boundary of a new phase in the evolution of *Homo sapiens* (Rolland, 2004; Bergström et al., 2021), and it is likely when the incremental evolution of protomusicality occurred (Killin, 2018).

The *Homo sapiens* is a pyrophilic primate (Parker et al., 2016). The human use of fire is very ancient and universal, and despite its dangerousness, children across cultures have an intrinsic curiosity about fire and achieve mastery of fire by middle childhood with little instruction (Fessler, 2006). This suggests that humans might have evolved learning mechanisms focused on controlling/maintaining and starting fire (Fessler, 2006). Paleolithic humans 170–150 ka ago optimally located the cave fireplace enabling maximum fire use and minimum exposure to smoke (Kedar et al., 2022). Fire provided our ancestors with heat, light, protection against predators and hostile conspecifics, smoke repellent of insects, aid in hunting and in preparing tasty, sterilized, preserved, and easily digestible cooked food, cremation of the dead, even improving flaking properties of stones, and aiding other tool manufacturing (Rolland, 2004; Coolidge and Wynn, 2006; Fessler, 2006; Brown et al., 2009; Wrangham, 2009; Dunbar and Gowlett, 2014). These benefits of habitual fire use set the stage for cognitive (Twomey, 2013), socioecological (Rolland, 2004; Dunbar and Gowlett, 2014), and cultural (Mithen, 2019) evolutionary change.

Therefore, the tree-to-ground nest transition, predation, and fire were recurrently enough to have converged and acted as selective pressures (Rolland, 2004; Coolidge and Wynn, 2006; Roebroeks and Villa, 2011; Twomey, 2013; Dunbar and Gowlett, 2014; Gowlett, 2016; Wrangham, 2017; Fruth et al., 2018; Samson, 2021; Hagen, 2022), expanding the ape-like flexible nocturnal niche in humans (Samson and Nunn, 2015; Tagg et al., 2018). The derived/efficient human sleep has allowed more nocturnal activities and higher exposure to nocturnal selective pressure (Samson and Nunn, 2015; Nunn et al., 2016; Nunn and Samson, 2018), which, I argue, could lead the ape-like protomusicality (including long-distance nocturnal vocalizations) to be refined into human musicality.

### 1.6. The challenging/opportunistic recurrent features of the night-time

Adaptive problems can impose “must-solve” challenges to survival and reproduction or offer beneficial opportunities to increase inclusive fitness (Lewis et al., 2017). The strength of each adaptive problem depends on its frequency and the magnitude of its impact on fitness (Lewis et al., 2017), as well as on its duration and how enduring it was throughout human evolution. Night-time happens every day (i.e., frequently and consistently), lasts roughly 12 h per day (i.e., long-lasting), and humans have been expanding into the nocturnal niche at least since the habitual use of fire (400–300 ka ago) (i.e., ancient/long-standing). The night-time exhibits a predictable selective landscape consisting of a collection of adaptive problems of varying magnitudes (e.g., predation and thermoregulation) (Fruth et al., 2018; Pozzi et al., 2022). Expanding on Varella et al. (2021) and Varella and Valentova (2023) and others (e.g., Putilov, 2014; Killin, 2017; Nowell, 2018), I propose that the six crucial specific and recurrent ancient adaptive challenges/opportunities faced by early hominins during awakening night-time were *lack of luminosity, low temperatures, regrouping to cook before sleep, dangerousness, peak tiredness, and concealment of identity.*

Those nocturnal adaptive challenges/opportunities could have, in combination, generated adaptive responses reusing, modifying, and interconnecting the preexisting variation in psychological traits, including protomusicality, that were reasonably suitable to solve each task. As a probable signature of the nocturnal selection, those improved preexisting psychological capacities might have coevolved and be phenotypically associated with the eveningness chronotype. The circadian rhythm of an individual’s psychophysiological well-functioning and intrinsic timing preferences for awakening and sleeping is conceptualized as the chronotype continuum and governed by internal clocks open to environmental cues (Vink et al., 2001; Preckel et al., 2011; Jones et al., 2019). Morning-oriented individuals wake up and go to sleep early while evening-oriented individuals wake up and go to sleep late (Vink et al., 2001; Preckel et al., 2011; Jones et al., 2019).

### 1.7. The lack of luminosity—why sound, loudness, repetition, and mnemonic effect?

Low luminosity impairs vision, creating a selection favoring hearing and night vision, leading to increased acoustic communication (Yoshida and Okanoya, 2005). Ontogenetically, initial auditory capacities could be improved following many night-time experiences. A comparable dynamic occurs in a compensatory way in early blind individuals, given that they have better spatial abilities (Collignon et al., 2006), auditory memory (Röder et al., 2001), and emotional activation to auditory stimuli (Klinge et al., 2010) and rely more on acoustic perception to evaluate a potential romantic/sexual partner (Sorokowska et al., 2018) than sighted counterparts. These auditory expansions throughout ontogeny were probably further intensified while living in dark caves. Interestingly, parietal paleoart is habitually found in places of caves, cliffs, and canyons with higher acoustic resonance, echoes, and reverberation (Waller, 2002; Reznikoff, 2008), indicating that ancient humans in the dark were very focused on sounds and gathering around places with exquisite acoustics.

Phylogenetically, a recent investigation including 1,800 tetrapod species showed that the origin of acoustic communication is strongly associated with a nocturnal lifestyle (Chen and Wiens, 2020). Living longer under low luminosity likely selects for higher acoustic communicative abilities, so that various nocturnal species independently evolved similar increased acoustic communication (Chen and Wiens, 2020). This adaptive convergence gives a strong indication that the improvement upon the ape-like protomusicality into the full human musicality (and even human language) likely occurred under nocturnal socioecological pressures.

Acoustic communication is flexible, fast, and long-ranged, allows sending complex and long messages, and overcomes obstacles. However, it is costly because it has a high degradation rate, is subject to interference, attenuation, and distortion with distance, and gives off the localization of the emitter allowing interception by natural enemies (Grier and Burk, 1992; Römer, 2001). The high diversity of species exhibiting nocturnal acoustic communication (Chen and Wiens, 2020) results in a noisier nocturnal environment (Römer, 2001). There are many strategies used across animal species to increase the signal-to-noise ratio (i.e., to avoid the background noise creating sound interference and distortion). The increases in signal amplitude and repetition are important and disseminated strategies across species to boost signal-to-noise ratio (Römer, 2001). Interestingly, humans are among the loudest terrestrial animals (Jakobsen et al., 2021). Compared to speaking, singing is louder and requires more lung capacity and more muscle activity (Leanderson et al., 1987; Åkerlund and Gramming, 1994; Lindblom and Sundberg, 2014; Savage et al., 2015), and music allows much more repetition of motifs/phrases, song sections, and entire songs/repertoire (Fitch, 2006; Savage et al., 2015). The consequent high accepting/liking of repetition in music (Madison and Schiölde, 2017) might have, along with other attributes (Levitin, 2021), made music a good mnemonic device (Schulkind, 2009; Reagh et al., 2017), explaining the cross-cultural existence of knowledge songs (Levitin, 2008, 2021). A similar mnemonic effect is found in the repeating patterns of whale song (Guinee and Payne, 1988).

### 1.8. Low temperatures—why long-distance loud calls and group cohesion?

After sunset, there is a period of temperature inversion when the ground gets cold faster than the air above trapping the sound energy in a sequence of refraction downward and reflection upwards creating a ‘sound duct’, a window of opportunity for optimal long-distance sound transmission (Larom et al., 1997; Römer, 2001; Piel, 2018). For instance, the calling range of savanna elephants increases up to 3-fold in the 2 h between late afternoon and early evening in dry and low wind conditions (Larom et al., 1997). Chimpanzees’ twilight calls are more frequent in dry seasons than in wet seasons (Piel, 2018), and at night, they perform long-distance vocalizations twice more often than proximity calls (Pruetz, 2018). The evening optimal transmission period for long-distance communication has probably been a selective pressure on many species in the African Savanna, including the lions, which mostly vocalize during the evening/night, and many others in savanna-like ecosystems such as coyotes and wolves which are also crepuscular vocalizers (Larom et al., 1997). Even diurnal animals, such as most birds and insects, tend to concentrate their acoustic communication during the times of temperature inversion near sunrise and sunset, generating the dawn and dusk choruses (Römer, 2001). Thus, due to the early evening cold ground creating favorable conditions for loud long-distance calls (Larom et al., 1997), early hominins could have been selected to make use of this acoustic opportunity to increase their nocturnal long-distance communication, expanding upon the ape-like correspondent precursors. Furthermore, the warm air/smoke from the campfire could, in principle, help maintain, for a longer time the temperature inversion favoring long-call communication.

Ancient hominins increased their nocturnal vocal activities, but night-time creates the challenge of avoiding hypothermia. The use of fire and the use of clothing are important strategies for thermoregulation. However, the habitual use of fire only started approximately 400–300 ka ago (Rolland, 2004; Roebroeks and Villa, 2011; Scott and Hosfield, 2021), and the earliest indication of clothing dates only 170–120 ka ago (Toups et al., 2011; Hallett et al., 2021). Thus, before habitual use of fire and clothing, during awake nocturnal activities, early hominins needed to rely on maintaining enduring physical activity and increase close contact with conspecifics to cope with the cold night-time, as a social thermoregulation (IJzerman et al., 2015). Arguably, like the penguins, early hominins could have performed social thermoregulation by maintaining group cohesion (IJzerman et al., 2015), doing useful nocturnal social activities in close proximity, including vocalizations and movements such as proto-dances.

### 1.9. The opportunity of regrouping to cook before sleep—why call and response, song, group cohesion, and playfulness/entertainment?

In the dark, it is unpractical/difficult for humans to forage (*cf.* Nowell, 2018). Moreover, humans, as animals, need to rest and sleep. Thus, after fission into subgroups for daily foraging comes the evening multi-male multi-female fusion (Foley and Gamble, 2009). Around the sunset is the time for foraging subgroups to reunite to the safety of the collective sleeping site. This place of regrouping bears similarities with the great ape night-time land use: a well-chosen place near resources such as water or fruiting trees, and at a period of increased sociality and group cohesion (Anderson et al., 2019). The optimal long-distance sound transmission (Larom et al., 1997; Römer, 2001; Piel, 2018) could have been used to call group members to the nearest sleeping site, because, in Chimpanzees, long-distance calls facilitate fusion (Fedurek et al., 2014). This might be one reason for the existence of responsorial singing (i.e., call and response) in traditional music (Kubik, 2005; Brown, 2017).

Before the habitual use of fire, the place of regrouping of early homo (2 Ma ago) was probably a “core area”: A preferred, but not fixed, night-time place containing natural protective topographies (caves and hills) combined with artificially made protective barriers in an ecotone habitat (overlapping grasslands and woodlands) where a small extended family congregated to fulfill subsistence activities, socialize, and, in a more reserved part far from eating location, prepare the ground nests (Rolland, 2004; Carotenuto et al., 2016). The core area could have been protected by stone-throwing sentinels (Samson et al., 2017; Lombardo and Deaner, 2018). *Homo erectus* were skilled tool makers, gatherers, scavengers, and carnivorous, fully biped with increased mobility and dispersion pattern, so the core areas could have been distantly scattered along increased home ranges (Rolland, 2004; Foley and Gamble, 2009). *Homo erectus* were able to expand from Africa to Eurasia and Southeast Asia following large herbivores, courses of water, and avoiding predators (Carotenuto et al., 2016). The increased home ranges certainly augmented the difficulty of males to find and monopolize resources (which were dispersed, and prey are very mobile) and mates (who were dispersed, very mobile, and had kin in defense), which put them in a socioecological context with key elements (including the ‘sound duct’ of optimal long-distance transmission) of three-dimensional niches (water and air), favoring the evolution of elaborated acoustic displays *via* sexual selection (*cf.* Puts, 2010; Varella et al., 2017; Verpooten and Eens, 2021).

Moreover, given that *Homo erectus* were also less sexually dimorphic, and due to increased infant dependence, they were probably more (serial) monogamously oriented exhibiting biparental provisioning/care (Fisher, 1989; Eastwick, 2009) and grandmother provisioning/care (O’Connell et al., 1999; Bell et al., 2013). Indeed, social monogamy and small family-sized groups are consistent predictors of complex song in vocal communication across primates (Haimoff, 1986; Geissmann, 2000; Schruth et al., 2021), and the majority of duetting primates are nocturnal or live across great distances in visually unreachable environments (*cf.* Geissmann, 2000; Clink et al., 2020; Pozzi et al., 2022). Duetting songbirds also tend to be monogamous (Geissmann, 2000). The evolution of elaborated songs across species, such as baleen whales, seals, songbirds, and hummingbirds, is also related to small, less complex social groups interacting over large distances beyond visual reach (Verpooten, 2021; Verpooten and Eens, 2021; Vanderhoff and Bernal, 2022). Thus, it is possible that, before the habitual use of fire and its consequent increase in social complexity (Foley and Gamble, 2009), early *Homo* was already prone to singing (*cf.* Mithen, 2005; Savage P. E. et al., 2021) to defend/advertise the night-time territory and to attract/maintain mates (*cf.* Hagen and Bryant, 2003) as a result of the ongoing amplification of nocturnal long-distance elaborated vocalizations (*cf.* Geissmann, 2000).

After the habitual use of fire, ancient hominins most probably had a “home base,” which is a more fixed base for both day and night operations, near valuable resources and strategic ecotone settings, with marked multipurpose fire use, cooking and social eating, protection of juvenile, transmission of knowledge, and a more patterned spatial organization (Rolland, 2004; Foley and Gamble, 2009). In current hunter-gatherer societies, the overnight camp has 35–70 individuals (Gowlett et al., 2012). After day-time fission foraging in transient sites, ancient hominins needed to find the way back to their home base, to process, cook, and share food, which takes time. The easily digestible cooked food increased caloric intake diminishing foraging time and enabling more time for evening socialization in a bigger group (Wrangham, 2009, 2017; Dunbar and Gowlett, 2014). There was a need for safe time-passing awake activities (Huron, 2001). During this forage-free time, the kin-based bigger group could engage in many social interactions (Nowell, 2018) such as childcare and play, information exchange, gossip, flirt, treat their wounds, prepare the ground sleeping nest, and guard the area against predators/enemies. The need to sleep near each other, to share cooked food, and to coordinate firewood gathering and keeping fire alight are important factors increasing in-group cohesion (Gowlett et al., 2012; Dunbar and Gowlett, 2014). In addition to domestic necessities, humans gained longer leisure time after habitual fire use (Killin, 2018). At least in contemporary societies, most leisure time in modern humans happens during the evening (Glorieux et al., 2010) and at home (Cushman et al., 2005). Thus, since musical activities are intrinsically motivated (Wang et al., 2018; Varella, 2021) and very popular leisure activity (McManus and Furnham, 2006; Varella, 2022), it is possible that musicality would be mostly expressed during ancestral evenings.

### 1.10. Dangerousness—why chills, creativity, in-group cohesion/outgroup competition, and anxiety modulation?

Despite the communal and comforting home base atmosphere, the night-time was, and still is, dangerous. Even baboons are more vulnerable to leopard attacks at night (Isbell et al., 2018). Hominin ancestors were preyed by a diversity of large diurnal and nocturnal carnivores, such as hyaenids and felids (Lee-Thorp et al., 2000; Barrett, 2005; Treves and Palmqvist, 2007; Hagen, 2022). In current night-times, humans in small-scale African societies still suffer from lion attacks (Packer et al., 2011), and in industrial societies, there are more personal contact crimes (Averdijk and Bernasco, 2015). In the dark, it is difficult to detect danger from predators and enemies, and as time passes, there are fewer bystanders awake who could promptly help in case of an attack (Jonason et al., 2013). These select for higher imagination and alertness toward harmful agents, openness to experience, conspicuous preventive defenses, social agglomeration, risk-taking/sensation-seeking, and forms of anxiety alleviation (Varella et al., 2021; Varella and Valentova, 2023).

The absence of visual stimuli alone upturns tension, uncertainty, and apprehension predisposing to the acoustic startle reflex (Grillon et al., 1997); nevertheless, it is not darkness alone, but the night that is most related to fear responses (Levos and Zacchilli, 2015; Li et al., 2015). Anxiety keeps the body in alert mode; thus, it is linked to insomnia (Oh et al., 2019). Interestingly, some musical passages related to peak emotional experiences and violation of expectations tend to trigger ‘chill’ experiences comprising heart racing, pupil dilation, and specially piloerection (Grewe et al., 2005; Laeng et al., 2016), which are old phylogenetic reactions among primates toward threatening (Mobbs, 2018; Kret et al., 2020) and cold situations (Chaplin et al., 2014), both nocturnal challenges.

The emotion of fear reorganizes our mindset strategically lowering the thresholds for the detection of danger. When there is a greater cost of missing a real threat than seeing a misapprehension of a hazard, evolution selects the less costly error leading to a higher false alarm rate (Nesse, 2001; Haselton and Nettle, 2006). When we are frightened, less evidence is needed to trigger the threat response, protecting against the cost of not perceiving a real threat (Tooby and Cosmides, 2008). Because of the nocturnal dangerousness, we might have evolved an overexcited imagination; a tendency to overestimate threats, purposes, and agency, particularly stemming from opponents (Barrett, 2005; Varella, 2018; Needham et al., 2022). Interestingly, in many species, there is the phenomenon of predator inspection, which is a contra-intuitive curiosity toward dangerous agents probably enabling better assessment of the situation and learning about the habits and behavioral patterns of predators (*cf.* Fishman, 1999). Hence, individual personality features, such as risk-taking and openness to experience (i.e., curiosity, fantasy, and esthetics), would improve responses to night-time dangers and mysteries, particularly in those less vulnerable individuals (i.e., adolescents and adults) who would have more to lose in terms of social networking, sexual opportunities, knowledge gathered by not socializing facing the nocturnal risks. Indeed, the evidence suggests that risk-taking and openness to experience are related to evening orientation and aspects of musicality. Evening-oriented individuals are risk-takers (Ponzi et al., 2014; Gowen et al., 2019), low on fear and caution (Ottoni et al., 2012), high on openness to experience (Randler et al., 2017), high on originality, open to culture and novelties, curious and well-informed (Cavallera and Giampietro, 2007), and creative and intuitive (Diaz-Morales and Escribano, 2013). Individuals open to experience are more musically/artistically/esthetically oriented (Rawlings et al., 2000; Feist and Brady, 2004; Gjermunds et al., 2020). Risk-taking/sensation-seeking/impulsivity is high in individuals with increased emotionality toward music (Roberts et al., 1998), in male individuals exhibiting more attractive dance movements (Hugill et al., 2011), and in individuals who prefer rock/heavy music (Little and Zuckerman, 1986; Arnett, 1992). Finally, young pop musicians are twice as likely to die of violent death (Kenny and Asher, 2016).

Moreover, the necessity to solve new practical problems during the night and the increased rate of REM sleep (i.e., indicative of dreaming) also converged possibly leading to higher creativity (Coolidge and Wynn, 2006; Samson and Nunn, 2015). Creativity is positively related to musicality and visual arts (Ismail et al., 2020; Sotiropoulos and Anagnostouli, 2021). Evidence for a nocturnal overexcited imagination which corroborates the *Nocturnal Evolution of Human Musicality and Performativity Theory* ranges from contemporary industrial societies, traditional societies, and prehistory. In industrial societies, music composers (Gjermunds et al., 2019), art students (Wang and Chern, 2008), and visually creative individuals (Giampietro and Cavallera, 2007) are indeed more evening-oriented. In traditional society, there is the highest frequency of storytelling in the evening (81% vs. 6% of day conversations) with background music around the campfire (Wiessner, 2014). Even some recent prehistoric findings support the nocturnal theory, such as some esthetically engraved plaquettes from 23 to 14 ka ago, which were likely appreciated by early humans close to the night-time firelight (Needham et al., 2022), and possible ‘shadow play’ storytelling 6.8–5.2 ka ago (Ahola and Lassila, 2022).

Combined with fire, human sounds, particularly when collective, loud, and rhythmically coordinated, which maximizes the salience of the collective broadcast (Keller et al., 2017), might have frightened ancestral predators as a conspicuous audio-visual intimidating display for night-time defense (Jordania, 2011; Hagen, 2022). Large carnivores avoid places with human voices (Suraci et al., 2019). When vervet monkeys give ‘leopard’ alarm calls in their sleeping site, most often near dusk and dawn, nearby leopards indeed move away (Isbell and Bidner, 2016). There is a Scandinavian pastoral musical tradition sung by women over long distances that is suggested to have long been used to coordinate the grazing of livestock during day-time and to frighten predators, such as bears and wolves, with horns and lures when women returned home in the evening (Ivarsdotter, 2004). In addition to the predators, ambush tactics and night-time attacks have long been modes of warfare (Sheldon, 2012; Redmond, 2016). The optimal long-distance sound transmission during the evening (Larom et al., 1997; Römer, 2001; Piel, 2018) could increase the range and effect of acoustic displays for predator and enemy deterrence, by canalizing loud calls (*cf.* Hagen and Hammerstein, 2009). According to Hagen and Bryant (2003), under night’s poor visibility, synchronized musical displays might have distantly signaled coalition size and formidability, frightening enemies (*cf.* Jordania, 2011; Mehr et al., 2021; Hagen, 2022). Indeed, evening-oriented men are high on intrasexual competitiveness (Ponzi et al., 2015a). Furthermore, the ‘many eyes’/‘security in numbers’ principles (Quenette, 1990; Musharbash, 2013) could have been selected for higher social nocturnal agglomeration together with low temperatures and the necessity to regroup before sleep. The ‘better safe than sorry’ principle might have led to exaggerated and frequent defensive rituals (Boyer and Linard, 2006; Eilam et al., 2011). Interestingly, musicality’s role in maintaining and promoting prosociality (Kirschner and Tomasello, 2010) and interpersonal social bonds (Boer et al., 2011; Savage P. et al., 2021) might indicate that it was selected under nocturnal selective pressures when the group is already on fusion mode, not fission (*cf.* Nowell, 2018).

The dangerousness of night-time also selects for anxiolytic activities: At the end of the day, ancient hominins needed to feel safe and relaxed enabling sleep. Anxiety often results in rituals (Eilam et al., 2011). The patterned repetitions of rituals have tension-reducing and comforting effects necessary to cope with dangerous/unpredictable environments (Dissanayake, 2008; Tonna et al., 2020). The very predator and enemy deterrence effects of music could attest/explain its anxiolytic properties. Among other health benefits, music demonstrably alleviates pain and diminishes stress and anxiety (Finn and Fancourt, 2018), particularly in social contexts (Linnemann et al., 2016). Indeed, late at night individuals prefer to listen to relaxing music (Park et al., 2019; Heggli et al., 2021). Since children are most vulnerable to nocturnal predation and infanticide, they mostly need lullabies, rocking, and bedtime stories for mood regulation and relaxation. An important complement to Savage P. et al. (2021) and Mehr et al. (2021) is that the parent–offspring bond is particularly challenged during bedtime to the point that the children need to be put to sleep by requiring costly displays of all-night parental protection strengthening their bond in order to relax and fall asleep. In fact, music has an important sleep-promoting role for infants and adults (Feng et al., 2018; Akkermann et al., 2021), explaining the existence of lullaby songs (Mehr et al., 2018, 2019), and a nightly bedtime routine is beneficial to children’s healthy sleep and wellbeing leading to satisfactory development (Mindell and Williamson, 2018).

### 1.11. Peak tiredness—why succumb to pleasurable and emotional stimuli?

At night-time, individuals are most tired. Tired individuals self-regulate less, thus easily succumb to immediate pleasures (Millar et al., 2019). Cravings occur mostly at night (Piasecki et al., 2011; Sevincer et al., 2016). Moreover, during off-peak periods in alertness and energy reserves, humans increase emotional reactivity (Tucker et al., 2012). Evening-oriented individuals have lower self-control (Digdon and Howell, 2008), lower emotional control, coping, and volition, but more emotional sensitivity (Ottoni et al., 2012), are impulsive (Cross, 2010), and hedonistically present-oriented (Nowack and Van Der Meer, 2013). The lower self-control, increased emotionality, immediacy, and hedonism in tired individuals at night-time might have further enhanced the ancestral appeal of recreational musical activities (*cf.* Nowell, 2018) and manipulation (De Tiège et al., 2021), given that music activates cerebral pleasure centers (Peretz, 2006), is among the most pleasurable activities (Dubé and Le Bel, 2003), and stimulates emotional reactions (Peretz, 2006; Altenmüller et al., 2013; Nowell, 2018).

### 1.12. Concealment of identity—why adolescence, mating and sexual benefits, complexity/elaboration, and mutual ornamentation?

Darkness partially conceals one’s identity providing a sense of privacy and anonymity, disinhibiting behavior usually repressed due to social desirability concerns (Zhong et al., 2010; Hirsh et al., 2011). Darkness improves individual autonomy by allowing control of emitting flow of information and external interferences (Klopfer and Rubenstein, 1977), lowering the risk for social embarrassment and reputational damage. Thus, by enabling high disinhibition, social proximity, emotional sensitiveness, risk-taking, hedonism, entertainment, creativity, and low self-control, the expanded nocturnal niche has favored an intense peruse of sexuality goals. As humans are among the rare animal species exhibiting indirect/covert courtship (Gersick and Kurzban, 2014) and private sexual intercourse (Ben Mocha, 2020), possibly because of the third-party costs (a jealous partner/competitor intercepting) among other reasons, it makes sense that humans lose their virginity at night (Barak et al., 1997) and exhibit the major peak of sexual activity at night, after 21:00 (Refinetti, 2005; Jankowski et al., 2014). Moreover, evening-oriented individuals tend to be single (Maestripieri, 2014), be more flirtations later in the day and have more sexual partners (Gunawardane et al., 2011), lose virginity early in life and have a short-term relationship orientation (Kasaeian et al., 2019), be more inclined to casual sex (Cross, 2010; Piffer, 2010; Matchock, 2018; Díaz-Morales et al., 2019), and have a fast life-history strategy (Ponzi et al., 2015b; Marvel-Coen et al., 2018).

This elevated night-time mating action amplified sexual selection pressures acting on human sleep (Piffer, 2010), chronotype (Putilov, 2014), and, possibly, musicality (Putilov, 2014; Varella and Valentova, 2023) and other artistic propensities (Putilov, 2014). Importantly, sexual selection is not only about male-biased sex differences (Varella et al., 2014, 2017; Bowling et al., 2021; Merker, 2021; Verpooten and Eens, 2021). As human exhibit mutual mate choice (Stewart-Williams and Thomas, 2013a,b), parental influence over mate choice (Apostolou, 2007), multifunctional signals (Berglund et al., 1996; De Block and Dewitte, 2007; De Tiège et al., 2021), multiple/multimodal cues (Candolin, 2003), self-presentation modification (Valentova et al., 2021), and indirect/covert flirtation (Gersick and Kurzban, 2014), the boundaries between sexual and social selection tend to get blurred (*cf.* West-Eberhard, 2014; Winegard et al., 2018; Crespi et al., 2022). Socialization at the ancestral home base, containing self-promotion and prestige/ornamented competition (Varella et al., 2017), could also function as a nocturnal mixed-sex lekking ground, where adolescent and young adults exhibit their desirable qualities to attract, compete for, and defend/maintain romantic partners (Dunbar et al., 1997; Piffer, 2010; Putilov, 2014). Miller (2000) reasoned that youngsters dancing all night at rave parties paint a somewhat accurate picture of how our ancestors engaged with their music (*cf.* Jenkinson et al., 2014). Adolescents, in the prime of mating competition, are the most evening-oriented humans (peaking around age 19, Randler, 2016; Fischer et al., 2017). They listen to and enjoy music the most, give more importance to music, and listen to music in many contexts (Bonneville-Roussy et al., 2013). Adults mostly remember and self-identify with music heard during adolescence and young adulthood (Jakubowski et al., 2020; Loveday et al., 2020).

Many recognized locations/contexts for short-term mating are shifted to night-time and contain loud music, e.g., nightclubs, dance clubs, bars, (fraternity) parties, concerts/festivals, and weddings (Jonason et al., 2015), where seduction culminates into the high synchrony of couple’s body movements (Brak-Lamy, 2015). Compared to afternoon, music-listening preferences after sunset are focused on increasingly higher tempo and danceability (Heggli et al., 2021). Listening to music increases the desire to date someone (Marin and Rathgeber, 2022), dancing with someone is ranked as an effective flirting tactic for short-term mating (Wade et al., 2021; Kennair et al., 2022), and the music-induced coupling of body sway predicts long-term romantic interest (Chang et al., 2021). The effects of darkness in reducing social embarrassment (Hirsh et al., 2011) might have encouraged individuals to overcome shyness/face-saving tendencies (Garland and Brown, 1972) to overtly perform (sing, dance, and tell stories) in front of others increasing the odds of attracting/maintaining a partner. Indeed, compared to the entrance, there is a 50% increase in coupled individuals exiting the nightclub, which is dark and musical (Mannion et al., 2009). Thus, musicality and other performances could have entered the playful human mating games (De Block and Dewitte, 2007; Moraes et al., 2022).

The complexity of social interactions, the conflicts, and alignments of interests inherent to sexual reproduction and animal communication (senders and receivers) create arms races and runaway dynamics in which the need to impress and persuade in competitive ornamentation leads to an increase in complexity, elaboration, and embellishments of songs (Miller, 2000; De Block and Dewitte, 2007; Varella et al., 2011, 2017; Keller et al., 2017; Mehr et al., 2021; Verpooten and Eens, 2021; Crespi et al., 2022). Indeed, in birds, song complexity is positively related to reproductive success (Soma and Garamszegi, 2011). Women in their fertile phase prefer composers of complex music for short-term relationships (Charlton, 2014) and dance seductively (Miller et al., 2007). Mating motives increase creativity in both sexes (Griskevicius et al., 2006). Overall, the increased nocturnal sexual selection can help explain the existence of serenade, seduction, love, and dance songs (e.g., Levitin, 2008; Lehman et al., 2009; Mehr et al., 2018, 2019), the diversity and appeal of love songs (Werner and Todd, 1997; Knobloch and Zillmann, 2003), the prevalence of sexual/reproductive messages in popular songs across genres (Dukes et al., 2003; Hobbs and Gallup Jr., 2011), and the sexual/relationship benefits of musicality (Van Bohemen et al., 2018; Micoogullari et al., 2021; Marin and Rathgeber, 2022).

Sexual selection might have influenced musicality early in hominin evolution (Garfinkel, 2018; Verpooten and Eens, 2021). Before the habitual use of fire, the socioecological context of *Homo erectus* favoring the emergence of elaborated songs (i.e., difficulty in monopolizing resources/partners, high mobility, vast home ranges, small social groups, and optimal context for long-distance sound transmission) is fairly disseminated across singing species, and serving sexual/social selection: mate attraction/maintenance and territory advertising/defense (Searcy and Andersson, 1986; Yoshida and Okanoya, 2005; Mikula et al., 2021; Schruth et al., 2021; Verpooten and Eens, 2021), as Darwin first proposed Darwin (1871). Social monogamy is another socioecological context favoring the evolution of complex song across primates (Schruth et al., 2021) and mutual display by duetting (Geissmann, 2000; Yoshida and Okanoya, 2005). In birds, when biparental care, leading to social monogamy with low rates of extra-pair paternity, is combined with cooperative defense of territories/breeding sites, there is the evolution of ornamentation in both sexes (Tobias et al., 2012). Mutual ornaments also function as social armaments in both sexes of birds (Tobias et al., 2011) and humans (De Block and Dewitte, 2007; Varella et al., 2017; Mafra et al., 2020). Interestingly, the combination of grandmother alloparental care (O’Connell et al., 1999; Bell et al., 2013) and children and elderly individuals sleeping early (Randler, 2016; Fischer et al., 2017) can effectively free women from the parental burden of late evening, enabling them to allocate efforts into mating attraction and intrasexual competition. In cooperative breeding birds, female ornamentation is indeed increased, decreasing sexual dimorphism (Dale et al., 2015). Thus, sexual selection in humans under monogamy, mutual mate choice, and alloparenting might have favored musical displays in both sexes, with an increased role of attraction and ornamental competition in women (Fisher and Candea, 2012; Varella et al., 2017, 2022) in comparison with other non-monogamous species without alloparental care.

Indeed, both men and women tend to have basically the same musical capacities with some minor distinctions on how musicality is expressed (*cf.* Mosing et al., 2015; Bertolo et al., 2023). Women are slightly better at recognizing familiar melodies (Miles et al., 2016), at mistuning perception (Bertolo et al., 2023), at music achievement (Mosing et al., 2015), appreciating more music (Hagen and Bryant, 2003; Varella et al., 2010), liking to sing more than men (Varella et al., 2010; De Moor et al., 2013; Yan et al., 2021); even the complexity of songs across primates is female-biased (Schruth et al., 2021). Compared to women, men tend to enjoy playing musical instruments more (Varella et al., 2010), tend to be slightly better in rhythmic discrimination task (Mosing et al., 2015; Bertolo et al., 2023), and slightly better in melodic and pitch discrimination tasks (Mosing et al., 2015); cross-culturally, music performers tend to be male (Savage et al., 2015; Mehr et al., 2018, 2019). Given that lower sound frequencies travel longer distances (Larom et al., 1997) and that non-human primates use low frequency for long-distance loud calls (Mitani and Stuht, 1998), there is a possibility that ancestor male hominins sang together to attract exogamic females traveling nearby (Merker, 1999, 2021) using the evening ‘sound duct’ (Delgado, 2006). Interestingly, a majority of adult humans fantasize about sex in traveling contexts (Lehmiller, 2018).

Another factor that can lead to mutual ornamentation is assortative mating (Tobias et al., 2012). The exaggeration of costly signaling can evolve under perfect monogamy through mutual mate choice and assortative mating (Hooper and Miller, 2008). In fact, assortative mating occurs among individuals similar in vocational interests (Etzel et al., 2019; Banov et al., 2022), playing musical instruments and singing (Bongard et al., 2019), musical and artistic talents (De Moor et al., 2013), creative achievements (Kaufman et al., 2016), sexual strategies (Penke and Asendorpf, 2008), and chronotype (Randler and Kretz, 2011), but see Horwitz et al. (2023). In addition, couples with corresponding chronotypes are more sexually active (Jocz et al., 2018) and sexually satisfied than mismatched couples (Sprajcer et al., 2022). Furthermore, assortative mating, which leads to the evolution of trait covariation (De Moor et al., 2013; Conroy-Beam et al., 2019), may have created the phenotypic relationships between the eveningness chronotype and musicality. Indeed, musicians and composers tend to be evening-oriented (Gjermunds et al., 2019; Wright and Palmer, 2022), and there is a genetic correlation between the eveningness chronotype, more accurate capacity for musical beat synchronization, and exposure to loud music (Niarchou et al., 2022).

## 2. Discussion

Why did musicality evolve? This “why” question has two aspects to be unpacked (*cf.* Dennett, 2017). The “how come that musicality evolved?” question requires a plausible step-by-step phylogenetic process narrative connecting ancestral ape-like protomusicality with human musicality. The “what reasons made musicality evolve?” question requires a probable combination of specific recurring selective pressures fitting most fitness-enhancing functions/roles of human musicality. The *Nocturnal Evolution of Human Musicality and Performativity Theory* (NEHMPT) tackles both aspects. It relies on interconnecting paleoanthropological phylogenetic patterns with a detailed evolutionary psychological analysis of recurrent nocturnal ancestral challenges/opportunities and correspondent outcomes, and with cross-species adaptive convergences, which offers a strong indication of adaptation (McGhee, 2011; Currie, 2012; Oikkonen et al., 2016). This newly proposed nocturnal theory is an extension and interconnection of previous related attempts that relate the evolution of musicality with night/eveningness (*cf.* Jordania, 2011; Dunbar and Gowlett, 2014; Putilov, 2014; Killin, 2017; Nowell, 2018; Varella et al., 2021; Varella and Valentova, 2023); it integrates many adaptive explanations/proposed proper functions for human musicality (e.g., parenting, group-cohesion, territorial/antipredator defense, and sexuality/romance) under the same umbrella theory. It also integrates ecological and social factors in shaping human musicality (*cf.* Schruth et al., 2021).

The possibility of a nocturnal evolution of human musicality does not imply that *all* humans should be evening-oriented nor that musical activities should *only* be found after sunset. Acoustic communication is often retained in lineages that evolved diurnality (Chen and Wiens, 2020), and mammals, in general, have retained many nocturnal features from the nocturnal bottleneck of the time of dinosaurs (Gerkema et al., 2013). Humans listen to music all day, but out of the five daily time blocks of music-listening preferences, three occur after sunset (evening, night, and late night; Heggli et al., 2021). I have provided evidence that musicality has a nocturnal bias. Even the music-listening effects on tennis players’ cognitive abilities are stronger during the evening (Jarraya and Jarraya, 2019). The evolutionary roots of this nocturnal bias are the focus of this proposal. I argue that the ancestral evening eco-social fusion context contained most of the key adaptive problems that musicality is suited to help solving. Throughout hominin evolution, there was an expansion of activities into a nocturnal niche, recurrently exposing humans (less vulnerable humans more than others) to enduring nocturnal challenges/opportunities impacting inclusive fitness: *lack of luminosity, low temperatures, regrouping to cook before sleep, imminent dangerousness, peak tiredness, and concealment of identity.* Despite being mostly active during the day, as humans became a more evening-inclined species in comparison with closely related apes, these high-frequency, long-lasting, and ancient night-time features might have selected together with the evening orientation, a propensity to acoustic communication, to be more alert and imaginative, gregarious, risk-taking and novelty-seeking, prone to anxiety modulation, hedonistic, and promiscuous and disinhibited, particularly in adolescents and adults, which in turn coevolved with protomusicality in different nocturnal contexts leading to the multifunctional human musicality. Those night-time features are independent from musical cognition and acted in combination as a coherent nocturnal selective landscape (i) directly promoting the enhancement of aspects of ape-like protomusicality into human musicality (e.g., darkness and temperature inversion promoting more long-distance acoustic communication, loudness, and repetition), (ii) selecting many psychological tendencies that coevolved with musicality (e.g., chronotype, openness to experience, and creativity), and (iii) creating the many co-occurring situational contexts to which musicality functionally adjusted (e.g., knowledge transference, recreation, parenting, group-cohesion, territorial/antipredator defense, infant-directed singing, and sexuality/romance).

This nocturnal theory does *not* invalidate/exclude other seriously proposed evolutionary theories of human musicality. It reorganizes and interconnects previous attempts into a parsimonious, overarching, and comprehensive framework. Instead of the final word, the current version of this nocturnal theory is the beginning of explorations of its heuristic value in generating new discoveries and connecting findings. For instance, it has been shown that music listening helps childbirth (Finn and Fancourt, 2018; McCaffrey et al., 2020), and given how evolutionarily problematic human delivery is, posing a survival problem to the mother and the newborn (Dunsworth and Eccleston, 2015), this might be considered another candidate adaptive effect of musicality. The nocturnal theory would predict that if there is any evolutionarily relevant activity recurrently happening during night-time, it is possible that musicality might have coevolved with it and be exapted to benefit the activity, in a process of cooptation by temporal juxtaposition. Indeed, it has been shown that non-human primates and humans tend to spontaneously give birth mostly during the night and early morning (Honnebier and Nathanielsz, 1994; Martin et al., 2018; Macfarlane et al., 2019; Declercq et al., 2023). The nocturnal onset of childbirth might be more temperature efficient (McFarland et al., 2022), free from insects and diurnal predators, and surrounded by more supporting conspecifics, despite vulnerability to nocturnal predators. Thus, activities that promote cohesion and deter predators decreasing pain, danger and anxiety would greatly benefit human childbirth; musical pain, activities seem to do so.

The *Nocturnal Evolution of Human Musicality and Performativity Theory* does *not* explain every single musical feature or underlying proximate mechanisms. Instead, it offers a plausible evidence-based scenario in which some important features (i.e., loudness, repetitiveness, call and response, chills, playfulness, complex ornamentation/virtuosity, and duets/chorus) and functions (i.e., long-distance communication, mnemonic device, dyadic and social bonds, leisure, territoriality, antipredator defense, emotional and mood regulation, sexuality, and childbirth) of musicality fit together. Furthermore, it does not suggest that *only* musicality was selected during the gradual expansion into the nocturnal niche. There are many non-exclusive strategic routes to reproductive success (De Block and Dewitte, 2007; Varella et al., 2017, 2022; Winegard et al., 2018). Similar to musicians, art students (Wang and Chern, 2008) and visually creative individuals (Giampietro and Cavallera, 2007) are also evening oriented. Thus, the nocturnal selective landscape is also relevant to the evolution of other forms of performing arts and performative entertainment using voice, such as humor, drama, and storytelling (Putilov, 2014; Wiessner, 2014; Lauer, 2022), including those forms focused on night-time horror/thriller stories (Clasen, 2012). Because of the firelight, visual arts by the fire (Needham et al., 2022) or in places with extraordinary acoustic properties (Waller, 2002; Reznikoff, 2008), including ‘shadow play’ storytelling (Ahola and Lassila, 2022) and fire-walking rituals (Xygalatas, 2015), are expected to be included in the scope of the *Nocturnal Evolution of Human Musicality and Performativity Theory*. This multimodal inclusive theoretical extension of the nocturnal theory might also help explain and integrate aspects of the proposed adaptive functions for visual arts (Power, 1999; Coe, 2003), literary arts and fiction (Gottschall, 2012; Smith et al., 2017; Dubourg and Baumard, 2022), language (Dunbar, 2004; Masataka, 2009; Dunbar and Gowlett, 2014), religiosity (Bulbulia, 2004; Sterelny, 2018), and popular culture (Salmon, 2018). Finally, even the cognitive niche hypothesis (Jonason et al., 2013) and eveningness epidemiological liability hypothesis (Varella et al., 2021) can be integrated into the *Nocturnal Evolution of Human Musicality and Performativity Theory* because the main nocturnal factors stressed by each hypothesis are inherent part among the night-time challenges/opportunities substantiating this nocturnal theory.

### 2.1. Future research

The relative lack of evidence connecting musicality with chronobiology/chronopsychology is a limitation. Some parts of the argument are still speculative in nature and need to be further developed and tested. The empirical support offered for this proposal still needs to be cross-culturally replicated. The current version of the nocturnal theory still needs further testing. Many questions remain unanswered. Evolutionary musicology should begin investigating the night following archeology (Gonlin and Nowell, 2017). How far can a singing group be heard or interact with other groups during the evening? Anthropologists studying traditional societies should try to determine using microphones how far a group sings can be heard under evening versus day-time conditions. Is there a nocturnal bias to sing loud, prefer repetitiveness, get the musical chills, compose, maintain a rhythm, perform, and appreciate music? Are the group cohesion effects and the anxiety-alleviating effects of music more pronounced at night? Are the effects of music on attractiveness evaluation and sexual excitement more pronounced in darker/nocturnal contexts? Music cognition researchers should try to compare the musical capacities and performances of the same individuals during day- and night-time conditions, controlling for other non-musical tasks or activities. Are nocturnal feline predators scared by group vocal music? Zoologists should try to investigate using playback whether big carnivores are attracted or repelled by human vocal group music.

The current theoretical proposition could be empirically refuted if the phenotypic and the genetic correlation between musicality and eveningness orientation is not replicated cross-culturally, or if the differences in musicality of adolescents and adults compared to youngsters and elderly are not replicated. It also could be refuted if future studies find no nocturnal bias on human musicality (production, appreciation, and motivation), particularly in adolescents and adults, and if future studies find no nocturnal bias for the effects and functions of musicality (i.e., long-distance communication, mnemonic device, dyadic and social bonds, leisure, territoriality, antipredator defense, emotional and mood regulation, sexuality, and childbirth). The current proposition offers precise and new ways of empirically testing human musicality and other performances.

## 3. Conclusion

The *Nocturnal Evolution of Human Musicality and Performativity Theory* (NEHMPT) argues that it is not a coincidence that humans are more nocturnal and musical than chimpanzees; that musicians, composers, and individuals with better beat synchronization capacities are evening-oriented; that most music-listening contexts are nocturnal; that adolescents, who are the most evening-oriented humans, enjoy more music; and that tribal nocturnal social activities around the campfire involve eating, singing, dancing, storytelling, and rituals. It presents convergent evidentiary support for the possibility that human musicality and performativity evolved mostly (but not only) under the influence of the nocturnal eco-social selective landscape. The nocturnal eco-social context is a common denominator among acoustic communication and the many functions, roles, and proposed adaptive functions of musicality. Musicality might function as a human’s night “long-distance vision”, night “sleeping bag”, night “social glue”, night entertainment and memory device, night “scarecrow” against predators/enemies, night anxiolytic and sleeping promoter, night “gala dress”, and night “midwife”. By integrating an evolutionary chronopsychology with evolutionary musicology, it is simultaneously possible to (i) explain why humans are more musical than the other great apes, (ii) explain the evolution of many aspects of musicality from independent principles, (iii) explain various features and functions of musicality, and (iv) integrate the different proposed ancestral adaptive values into an inclusive and coherent approach. Musicality is probably a multifunctional mental adaptation that evolved hand in hand with the many night-time adaptive problems. I hope to have shed some light on a missing piece of the adaptationist puzzle of human musicality.

## Data availability statement

The theoretical contributions presented in the study are included in the article, further inquiries can be directed to the corresponding author.

## Author contributions

MV searched the literature, conceptually analyzed, and organized the published evidence, conceived the main theoretical proposition, drafted, corrected, improved, and formatted the manuscript.

## References

[ref1] Ahola M. Lassila K. (2022). Mesolithic shadow play? Exploring the performative attributes of a zoomorphic wild reindeer (*Rangifer tarandus*) antler artefact from Finland. Time Mind 15, 167–185. doi: 10.1080/1751696X.2022.2098047

[ref2] Åkerlund L. Gramming P. (1994). Average loudness level, mean fundamental frequency, and subglottal pressure: comparison between female singers and nonsingers. J. Voice 8, 263–270. doi: 10.1016/S0892-1997(05)80298-X, PMID: 7987429

[ref3] Akkermann M. Akkaya U. C. Dermiel C. Pflüger D. Dresler M. (2021). Sound sleep: lullabies as a test case for the neurobiological effects of music. Behav. Brain Sci. 44:E96. doi: 10.1017/S0140525X20001259 34588040

[ref4] Akram U. Stevenson J. C. Gardani M. Akram A. Allen S. (2019). Psychopathy and chronotype disposition: the mediating role of depression. Heliyon 5:e02894. doi: 10.1016/j.heliyon.2019.e02894, PMID: 31844760 PMC6895668

[ref5] Altenmüller E. Kopiez R. Grewe O. (2013). “Strong emotions in music: are they an evolutionary adaptation?” in Sound-perception-performance. ed. Bader R., vol. 1 (Cham: Springer International Publishing), 131–156.

[ref6] Anderson J. R. Ang M. Y. Lock L. C. Weiche I. (2019). Nesting, sleeping, and nighttime behaviors in wild and captive great apes. Primates 60, 321–332. doi: 10.1007/s10329-019-00723-2, PMID: 30972523

[ref7] Ankel-Simons F. Rasmussen D. T. (2008). Diurnality, nocturnality, and the evolution of primate visual systems. Yearb. Phys. Anthropol. 51, 100–117. doi: 10.1002/ajpa.20957 19003895

[ref8] Apostolou M. (2007). Sexual selection under parental choice: the role of parents in the evolution of human mating. Evol. Hum. Behav. 28, 403–409. doi: 10.1016/j.evolhumbehav.2007.05.007, PMID: 35858444

[ref9] Arcadi A. C. Robert D. Boesch C. (1998). Buttress drumming by wild chimpanzees: temporal patterning, phrase integration into loud calls, and preliminary evidence for individual distinctiveness. Primates 39, 505–518. doi: 10.1007/BF02557572

[ref10] Arnett J. (1992). Musical preferences and reckless behavior among adolescents. J. Adolesc. Res. 7, 313–331. doi: 10.1177/074355489273003

[ref11] Averdijk M. Bernasco W. (2015). Testing the situational explanation of victimization among adolescents. J. Res. Crime Delinq. 52, 151–180. doi: 10.1177/0022427814546197

[ref12] Ball P. (2010). The music instinct: How music works and why we can't do without it. New York: Oxford University Press.

[ref13] Banov K. Krapic N. Kardum I. (2022). Do vocational interests matter for the selection of romantic partners? Evidence from variable-and couple-centered approaches. Appl. Psychol. 72, 697–717. doi: 10.1111/apps.12396

[ref14] Barak Y. Stein D. Ring A. Ticher A. Elizur A. (1997). Patterns of first intercourse: a survey among Israeli women. Biol. Rhythm. Res. 28, 36–41. doi: 10.1076/brhm.28.1.36.12972

[ref15] Barrett H. (2005). “Adaptations to predators and prey” in The handbook of evolutionary psychology. ed. Buss D. M. (New York, NY: Wiley), 200–223.

[ref16] Bell A. V. Hinde K. Newson L. (2013). Who was helping? The scope for female cooperative breeding in early homo. PLoS One 8:e83667. doi: 10.1371/journal.pone.0083667, PMID: 24367605 PMC3867437

[ref17] Ben Mocha Y. (2020). Why do human and non-human species conceal mating? The cooperation maintenance hypothesis. Proc. R. Soc. B. 287:1330. doi: 10.1098/rspb.2020.1330 PMC7575526 32752989

[ref18] Benítez-Burraco A. Nikolsky A. (2023). The (co)evolution of language and music under human self-domestication. Hum. Nat. 34, 229–275. doi: 10.1007/s12110-023-09447-1, PMID: 37097428 PMC10354115

[ref19] Berglund A. Bisazza A. Pilastro A. (1996). Armaments and ornaments: an evolutionary explanation of traits of dual utility. Biol. J. Linn. Soc. 58, 385–399. doi: 10.1111/j.1095-8312.1996.tb01442.x

[ref20] Bergström A. Stringer C. Hajdinjak M. Scerri E. M. Skoglund P. (2021). Origins of modern human ancestry. Nature 590, 229–237. doi: 10.1038/s41586-021-03244-5, PMID: 33568824

[ref21] Bertolo M. Müllensiefen D. Peretz I. Woolley S. C. Sakata J. Mehr S. (2023). Human music perception ability is not a sexually dimorphic trait. In Goldwater M. Anggoro F. K. Hayes B. K. Ong D. C. (Eds.), Proceedings of the Annual Meeting Cognition Science Society.

[ref22] Bispham J. C. (2009). Music's “design features”: musical motivation, musical pulse, and musical pitch. Music. Sci. 13, 41–61. doi: 10.1177/1029864909013002041

[ref23] Boer D. Fischer R. Strack M. Bond M. H. Lo E. Lam J. (2011). How shared preferences in music create bonds between people: values as the missing link. Personal. Soc. Psychol. Bull. 37, 1159–1171. doi: 10.1177/0146167211407521, PMID: 21543650

[ref24] Bongard S. Schulz I. Studenroth K. U. Frankenberg E. (2019). Attractiveness ratings for musicians and non-musicians: an evolutionary-psychology perspective. Front. Psychol. 10:2627. doi: 10.3389/fpsyg.2019.02627 31849756 PMC6895061

[ref25] Bonneville-Roussy A. Rentfrow P. J. Xu M. K. Potter J. (2013). Music through the ages: trends in musical engagement and preferences from adolescence through middle adulthood. J. Pers. Soc. Psychol. 105, 703–717. doi: 10.1037/a0033770, PMID: 23895269

[ref26] Bowling D. L. Hoeschele M. Dunn J. C. (2021). Progress without exclusion in the search for an evolutionary basis of music. Behav. Brain Sci. 44:E97. doi: 10.1017/S0140525X20001466 34588062 PMC8485979

[ref27] Boyer P. Linard P. (2006). Precaution systems and ritualized behavior. Behav. Brain Sci. 29, 635–650. doi: 10.1017/S0140525X06009575, PMID: 17918647

[ref28] Brak-Lamy G. (2015). Heterosexual seduction in the urban night context: behaviors and meanings. J. Sex Res. 52, 690–699. doi: 10.1080/00224499.2013.8 24483605

[ref29] Brown S. (2000). “Evolutionary models of music: from sexual selection to group selection” in Perspectives in ethology. eds. Tonneau F. Thompson N. S. (New York: Plenum), 231–281.

[ref30] Brown S. (2017). A joint prosodic origin of language and music. Front. Psychol. 8:1894. doi: 10.3389/fpsyg.2017.01894 29163276 PMC5666296

[ref31] Brown K. S. Marean C. W. Herries A. I. Jacobs Z. Tribolo C. Braun D.. (2009). Fire as an engineering tool of early modern humans. Science 325, 859–862. doi: 10.1126/science.1175028, PMID: 19679810

[ref32] Brown S. Merker B. Wallin N. L. (2000). “An introduction to evolutionary musicology” in The origins of music. eds. Wallin N. L. Merker B. Brown S. (Massachusetts: MIT Press), 3–24.

[ref33] Bulbulia J. (2004). The cognitive and evolutionary psychology of religion. Biol. Philos. 19, 655–686. doi: 10.1007/s10539-005-5568-6, PMID: 37587943

[ref34] Candolin U. (2003). The use of multiple cues in mate choice. Biol. Rev. 78, 575–595. doi: 10.1017/S1464793103006158, PMID: 14700392

[ref35] Carotenuto F. Tsikaridze N. Rook L. Lordkipanidze D. Longo L. Condemi S.. (2016). Venturing out safely: the biogeography of Homo erectus dispersal out of Africa. J. Hum. Evol. 95, 1–12. doi: 10.1016/j.jhevol.2016.02.005, PMID: 27260171

[ref36] Cavallera G. M. Giampietro M. (2007). Morning and evening personality characteristics in a sample of young Italians. Percept. Mot. Skills 104, 277–286. doi: 10.2466/pms.104.1.277-286, PMID: 17450989

[ref37] Chang A. Kragness H. E. Tsou W. Bosnyak D. J. Thiede A. Trainor L. J. (2021). Body sway predicts romantic interest in speed dating. Soc. Cogn. Affect. Neurosci. 16, 185–192. doi: 10.1093/scan/nsaa093 32685965 PMC7812630

[ref38] Chaplin G. Jablonski N. G. Sussman R. W. Kelley E. A. (2014). The role of piloerection in primate thermoregulation. Folia Primatol. 85, 1–17. doi: 10.1159/000355007, PMID: 24192984

[ref39] Charlton B. D. (2014). Menstrual cycle phase alters women's sexual preferences for composers of more complex music. Proc. R. Soc. B Biol. Sci. 281:20140403. doi: 10.1098/rspb.2014.0403, PMID: 24759864 PMC4043099

[ref40] Chen Z. Wiens J. J. (2020). The origins of acoustic communication in vertebrates. Nat. Commun. 11:369. doi: 10.1038/s41467-020-14356-3 31953401 PMC6969000

[ref41] Clasen M. (2012). Monsters evolve: a biocultural approach to horror stories. Rev. Gen. Psychol. 16, 222–229. doi: 10.1037/a0027918

[ref42] Clayton M. (2016). “The social and personal functions of music in cross-cultural perspective” in Oxford handbook of music psychology. eds. Hallam S. Cross I. Thaut M.. 2nd ed (Oxford: Oxford University Press)

[ref43] Clink D. J. Tasirin J. S. Klinck H. (2020). Vocal individuality and rhythm in male and female duet contributions of a nonhuman primate. Curr. Zool. 66, 173–186. doi: 10.1093/cz/zoz035, PMID: 32440276 PMC7233616

[ref44] Coe K. (2003). The ancestress hypothesis: Visual art as adaptation. New Brunswick, NJ: Rutgers University Press.

[ref45] Collignon O. Renier L. Bruyer R. Tranduy D. Veraart C. (2006). Improved selective and divided spatial attention in early blind subjects. Brain Res. 1075, 175–182. doi: 10.1016/j.brainres.2005.12.079, PMID: 16460716

[ref46] Conroy-Beam D. Roney J. R. Lukaszewski A. W. Buss D. M. Asao K. Sorokowska A.. (2019). Assortative mating and the evolution of desirability covariation. Evol. Hum. Behav. 40, 479–491. doi: 10.1016/j.evolhumbehav.2019.06.003

[ref47] Coolidge F. Wynn T. (2006). The effects of the tree-to-ground sleep transition in the evolution of cognition in early Homo. Before Farm. 4, 1–18. doi: 10.3828/bfarm.2006.4.11

[ref48] Coon H. Carey G. (1989). Genetic and environmental determinants of musical ability in twins. Behav. Genet. 19, 183–193. doi: 10.1007/BF01065903, PMID: 2719622

[ref49] Crespi B. J. Flinn M. V. Summers K. (2022). Runaway social selection in human evolution. Front. Ecol. Evol. 10:894506. doi: 10.3389/fevo.2022.894506, PMID: 36619053

[ref50] CrossC. P. (2010). Sex differences in same-sex direct aggression and sociosexuality: the role of risky impulsivity. Evol. Psychol. 8, 779–792. doi: 10.1177/14747049100080041822947835

[ref51] Cross I. Morley I. (2002). “Music and evolution: the nature of the evidence,” In Proceedings of the 7th International Conference on Music Perception and Cognition, eds. Stevens C. Burnham D. McPherson G. Schubert E. Renwick J. (Adelaide: Causal Productions).

[ref52] Cross I. Morley I. (2008). “The evolution of music: theories, definitions and the nature of the evidence” in Communicative musicality. eds. Malloch S. Trevarthen C. (Oxford: Oxford University Press), 61–82.

[ref53] Croston R. Branch C. L. Kozlovsky D. Y. Dukas R. Pravosudov V. V. (2015). Heritability and the evolution of cognitive traits. Behav. Ecol. 26, 1447–1459. doi: 10.1093/beheco/arv088, PMID: 37700643

[ref54] Currie A. (2012). Convergence as evidence. Brit. J. Phil. Sci. 64, 763–786. doi: 10.1093/bjps/axs027

[ref55] Currie A. Killin A. (2016). Musical pluralism and the science of music. Eur. J. Philos. Sci. 6, 9–30. doi: 10.1007/s13194-015-0123-z, PMID: 37680502

[ref56] Cushman G. Veal A. J. Zuzamek J. (2005). Free time and leisure participation international perspectives. Wallingford, UK: CABI Publishing.

[ref57] Dale J. Dey C. J. Delhey K. Kempenaers B. Valcu M. (2015). The effects of life history and sexual selection on male and female plumage colouration. Nature 527, 367–370. doi: 10.1038/nature15509, PMID: 26536112

[ref58] Darwin C. (1871). The descent of man, and selection in relation to sex. 1st 2 London: John Murray.

[ref59] De Block A. Dewitte S. (2007). Mating games: cultural evolution and sexual selection. Biol. Philos. 22, 475–491. doi: 10.1007/s10539-006-9041-y

[ref60] De Moor M. H. M. Roeling M. P. Boomsma D. I. (2013). “Creativity and talent: etiology of familial clustering” in Neuroscience of creativity. eds. Bristol A. S. Vartanian O. Kaufman A. B. (Cambridge, Mass: MIT Press), 95–112.

[ref61] De Tiège A. Verpooten J. Braeckman J. (2021). From animal signals to art: manipulative animal signaling and the evolutionary foundations of aesthetic behavior and art production. Q. Rev. Biol. 96, 1–27. doi: 10.1086/713210

[ref62] Declercq E. Wolterink A. Rowe R. de Jonge A. De Vries R. Nieuwenhuijze M.. (2023). The natural pattern of birth timing and gestational age in the US compared to England, and the Netherlands. PLoS One 18:E0278856. doi: 10.1371/journal.pone.0278856 36652413 PMC9847908

[ref63] Delgado R. A. (2006). Sexual selection in the loud calls of male primates: signal content and function. Int. J. Primatol. 27, 5–25. doi: 10.1007/s10764-005-9001-4

[ref64] Dennett D. C. (2017). From bacteria to Bach and back: The evolution of minds. New York: W.W. Norton & Company.

[ref65] Diaz-Morales J. F. Escribano C. (2013). Circadian preference and thinking styles: implications for school achievement. Chronobiol. Int. 30, 1231–1239. doi: 10.3109/07420528.2013.813854, PMID: 24024592

[ref66] Díaz-Morales J. F. Jankowski K. S. Prokop P. Rahafar A. (2019). Sleep timing is linked to sociosexuality: evidence from German, polish, Slovak, and Spanish females. Time Soc. 28, 1272–1287. doi: 10.1177/0961463X18757390

[ref67] Diez-Martín F. Sánchez Yustos P. Uribelarrea D. Baquedano E. Mark D. F. Mabulla A.. (2015). The origin of the Acheulean: the 1.7 million-year-old site of FLK West, Olduvai Gorge (Tanzania). Sci. Rep. 5:17839. doi: 10.1038/srep17839 26639785 PMC4671088

[ref68] Digdon N. L. Howell A. J. (2008). College students who have an eveningness preference report lower self-control and greater procrastination. Chronobiol. Int. 25, 1029–1046. doi: 10.1080/07420520802553671 19005903

[ref69] Dissanayake E. (2008). If music is the food of love, what about survival and reproductive success? Mus. Sci. 12, 169–195. doi: 10.1177/1029864908012001081

[ref70] Drayna D. Manichaikul A. de Lange M. Snieder H. Spector T. (2001). Genetic correlates of musical pitch recognition in humans. Science 291, 1969–1972. doi: 10.1126/science.291.5510.1969, PMID: 11239158

[ref71] Duba J. D. Roseman C. (2012). Musical “tune-ups” for couples: brief treatment interventions. Fam. J. 20, 322–326. doi: 10.1177/1066480712449604

[ref72] Dubé L. le bel J. (2003). The content and structure of laypeople's concept of pleasure. Cogn. Emot. 17, 263–295. doi: 10.1080/02699930302295 29715723

[ref73] Dubourg E. André J. B. Baumard N. (2021). The evolution of music: one trait, many ultimate-level explanations. Behav. Brain Sci. 44:E98. doi: 10.1017/S0140525X20001156 34588022

[ref74] Dubourg E. Baumard N. (2022). Why and how did narrative fictions evolve? Fictions as entertainment technologies. Front. Psychol. 13:786770. doi: 10.3389/fpsyg.2022.786770, PMID: 35300163 PMC8921504

[ref75] Dufour V. Poulin N. Curé C. Sterck E. H. (2015). Chimpanzee drumming: a spontaneous performance with characteristics of human musical drumming. Sci. Rep. 5:11320. doi: 10.1038/srep11320 26080900 PMC4469965

[ref76] Dukes R. L. Bisel T. M. Borega K. N. Lobato E. A. Owens M. D. (2003). Expressions of love, sex, and hurt in popular songs: a content analysis of all-time greatest hits. Soc. Sci. J. 40, 643–650. doi: 10.1016/S0362-3319(03)00075-2

[ref77] Dunbar R. I. M. (2004). “Language, music, and laughter in evolutionary perspective” in Evolution of communication systems: A comparative approach. eds. Oller D. K. Griebel U. (Cambridge, MA: MIT Press), 257–274.

[ref78] Dunbar R. Gowlett J. (2014). “Fireside chat: the impact of fire on hominin socioecology” in Lucy to language: The benchmark papers. eds. Dunbar R. Gamble C. Gowlett J. (Oxford: Oxford University Press), 277–296.

[ref79] Dunbar M. Marriott A. Duncan C. (1997). Human conversational behavior. Hum. Nat. 8, 231–246. doi: 10.1007/BF02912493, PMID: 26196965

[ref80] Dunsworth H. Eccleston L. (2015). The evolution of difficult childbirth and helpless hominin infants. Annu. Rev. Anthropol. 44, 55–69. doi: 10.1146/annurev-anthro-102214-013918

[ref81] Eastwick P. W. (2009). Beyond the pleistocene: using phylogeny and constraint to inform the evolutionary psychology of human mating. Psychol. Bull. 135, 794–821. doi: 10.1037/a0016845, PMID: 19702384

[ref82] Eibl-Eibesfeldt I. (1989). Human ethology. New York: Aldine de Gruyter.

[ref83] Eilam D. Izhar R. Mort J. (2011). Threat detection: behavioral practices in animals and humans. Neurosci. Biobehav. Rev. 35, 999–1006. doi: 10.1016/j.neubiorev.2010.08.002, PMID: 20727909

[ref84] Etzel J. M. Lüdtke O. Wagner J. Nagy G. (2019). Similarity of vocational interest profiles within families: a person-centered approach for examining associations between circumplex profiles. J. Pers. 87, 593–606. doi: 10.1111/jopy.12418, PMID: 29999531 PMC7379732

[ref85] Fedurek P. Donnellan E. Slocombe K. E. (2014). Social and ecological correlates of long-distance pant hoot calls in male chimpanzees. Behav. Ecol. Sociobiol. 68, 1345–1355. doi: 10.1007/s00265-014-1745-4

[ref86] Feist G. J. Brady T. R. (2004). Openness to experience, non-conformity, and the preference for abstract art. Empir. Stud. Arts 22, 77–89. doi: 10.2190/Y7CA-TBY6-V7LR-76GK

[ref87] Feng F. Zhang Y. Hou J. Cai J. Jiang Q. Li X.. (2018). Can music improve sleep quality in adults with primary insomnia? A systematic review and network meta-analysis. Int. J. Nurs. Stud. 77, 189–196. doi: 10.1016/j.ijnurstu.2017.10.011, PMID: 29100201

[ref88] Fessler D. (2006). A burning desire: steps toward an evolutionary psychology of fire learning. J. Cogn. Cult. 6, 429–451. doi: 10.1163/156853706778554986

[ref89] Figueredo A. J. Black C. J. Patch E. A. Heym N. Ferreira J. H. B. P. Varella M. A. C.. (2021). The cascade of chaos: from early adversity to interpersonal aggression. Evol. Behav. Sci. 15, 231–250. doi: 10.1037/ebs0000241

[ref90] Fink B. Bläsing B. Ravignani A. Shackelford T. K. (2021). Evolution and functions of human dance. Evol. Hum. Behav. 42, 351–360. doi: 10.1016/j.evolhumbehav.2021.01.003, PMID: 37676911

[ref91] Finn S. Fancourt D. (2018). “The biological impact of listening to music in clinical and nonclinical settings: a systematic review” in The arts and the brain: Psychology and physiology beyond pleasure. eds. Christensen J. F. Gomila A. (Amsterdam: Elsevier B.V), 173–200. 10.1016/bs.pbr.2018.03.007 29779734

[ref92] Fischer D. Lombardi D. A. Marucci-Wellman H. Roenneberg T. (2017). Chronotypes in the US–influence of age and sex. PLoS One 12:E0178782. doi: 10.1371/journal.pone.0178782 28636610 PMC5479630

[ref93] Fisher H. E. (1989). Evolution of human serial pairbonding. Am. J. Phys. Anthropol. 78, 331–354. doi: 10.1002/ajpa.1330780303, PMID: 2929738

[ref94] Fisher M. L. Candea C. (2012). You ain’t woman enough to take my man: female intrasexual competition as portrayed in songs. J. Soc. Evol. Cult. Psychol. 6:480. doi: 10.1037/h0099238

[ref95] Fishman M. A. (1999). Predator inspection: closer approach as a way to improve assessment of potential threats. J. Theor. Biol. 196, 225–235. doi: 10.1006/jtbi.1998.0834, PMID: 10049617

[ref96] Fitch W. T. (2006). The biology and evolution of music: a comparative perspective. Cognition 100, 173–215. doi: 10.1016/j.cognition.2005.11.009, PMID: 16412411

[ref97] Fitch W. T. (2015). Four principles of bio-musicology. Philos. Trans. R. Soc. B 370:20140091. doi: 10.1098/rstb.2014.0091, PMID: 25646514 PMC4321132

[ref98] Foley R. Gamble C. (2009). The ecology of social transitions in human evolution. Philos. Trans. R. Soc. B 364, 3267–3279. doi: 10.1098/rstb.2009.0136, PMID: 19805433 PMC2781881

[ref99] Franklin M. S. Rattray K. Moore K. S. Moher J. Yip C. Jonides J. (2008). The effects of musical training on verbal memory. Psychol. Music 36, 353–365. doi: 10.1177/0305735607086044, PMID: 37649718

[ref100] Fruth B. Tagg N. Stewart F. (2018). Sleep and nesting behavior in primates: a review. Am. J. Phys. Anthropol. 166, 499–509. doi: 10.1002/ajpa.23373, PMID: 29989164

[ref101] Fukui H. Toyoshima K. (2023). Testosterone, oxytocin and co-operation: a hypothesis for the origin and function of music. Front. Psychol. 14:1055827. doi: 10.3389/fpsyg.2023.1055827 36860786 PMC9968751

[ref102] Garfinkel Y. (2018). The evolution of human dance: courtship, rites of passage, trance, calendrical ceremonies and the professional dancer. Camb. Archaeol. J. 28, 283–298. doi: 10.1017/S0959774317000865

[ref103] Garland H. Brown B. R. (1972). Face-saving as affected by subjects’ sex, audiences’ sex and audience expertise. Sociometry 35, 280–289. doi: 10.2307/2786623, PMID: 5033657

[ref104] Geissmann T. (2000). “Gibbon songs and human music from an evolutionary perspective” in The origins of music. eds. Wallin N. L. Merker B. Brown S. (Cambridge, MA: MIT Press), 103–123.

[ref105] Gerkema M. P. Davies W. I. L. Foster R. G. Menaker M. Hut R. A. (2013). The nocturnal bottleneck and the evolution of activity patterns in mammals. Proc. R. Soc. B 280:20130508. doi: 10.1098/rspb.2013.0508, PMID: 23825205 PMC3712437

[ref106] GersickA.KurzbanR. (2014). Covert sexual signaling: human flirtation and implications for other social species. Evol. Psychol. 12:305. doi: 10.1177/14747049140120030525299992

[ref107] Giampietro M. Cavallera G. M. (2007). Morning and evening types and creative thinking. Pers. Individ. Differ. 42, 453–463. doi: 10.1016/j.paid.2006.06.027, PMID: 31118949

[ref108] Gildersleeve K. Haselton M. G. Fales M. R. (2014). Do women’s mate preferences change across the ovulatory cycle? A meta-analytic review. Psychol. Bull. 140:1205. doi: 10.1037/a0035438, PMID: 24564172

[ref109] Gjermunds N. Brechan I. Johnsen S. Å. K. Watten R. G. (2019). Musicians: larks, owls or hummingbirds? J. Circadian Rhythms 17:4. doi: 10.5334/jcr.173 31118949 PMC6509950

[ref110] Gjermunds N. Brechan I. Johnsen S. Å. K. Watten R. G. (2020). Personality traits in musicians. Curr. Issues Personal. Psychol. 8, 100–107. doi: 10.5114/cipp.2020.97314, PMID: 37691808

[ref111] Glorieux I. Laurijssen I. Minnen J. van Tienoven T. P. (2010). In search of the harried leisure class in contemporary society: time-use surveys and patterns of leisure time consumption. J. Consum. Policy 33, 163–181. doi: 10.1007/s10603-010-9124-7

[ref112] Gonlin N. Nowell A. (2017). Archaeology of the night: life after dark in the ancient world. Boulder: University Press of Colorado.

[ref113] Gordon R. L. Fehd H. M. McCandliss B. D. (2015). Does music training enhance literacy skills? A meta-analysis. Front. Psychol. 6:1777. doi: 10.3389/fpsyg.2015.01777, PMID: 26648880 PMC4664655

[ref114] Gottschall J. (2012). The storytelling animal: How stories make us human. New York: Houghton Mifflin Harcourt.

[ref115] Gowen R. Filipowicz A. Ingram K. K. (2019). Chronotype mediates gender differences in risk propensity and risk-taking. PLoS One 14:E0216619. doi: 10.1371/journal.pone.0216619 31120931 PMC6532857

[ref116] Gowlett J. A. J. (2006). The early settlement of northern Europe: fire history in the context of climate change and the social brain. C. R. Palevol. 5, 299–310. doi: 10.1016/j.crpv.2005.10.008

[ref117] Gowlett J. A. J. (2016). The discovery of fire by humans: a long and convoluted process. Philos. T. R. Soc. B. 371:20150164. doi: 10.1098/rstb.2015.0164 PMC4874402 27216521

[ref118] Gowlett J. Gamble C. Dunbar R. (2012). Human evolution and the archaeology of the social brain. Curr. Anthropol. 53, 693–722. doi: 10.1086/667994, PMID: 37614563

[ref119] Grewe O. Nagel F. Kopiez R. Altenmüller E. (2005). How does music arouse “chills”? Investigating strong emotions, combining psychological, physiological, and psychoacoustical methods. Ann. N. Y. Acad. Sci. 1060, 446–449. doi: 10.1196/annals.1360.041 16597800

[ref120] Grier J. W. Burk T. (1992). Biology of animal behavior. St Louis: Mosby-Year Book.

[ref121] Grillon C. Pellowski M. Merikangas K. R. Davis M. (1997). Darkness facilitates the acoustic startle reflex in humans. Biol. Psychiatry 42, 453–460. doi: 10.1016/S0006-3223(96)00466-0, PMID: 9285081

[ref122] Griskevicius V. Cialdini R. B. Kenrick D. T. (2006). Peacocks, Picasso, and parental investment: the effects of romantic motives on creativity. J. Pers. Soc. Psychol. 91:63. doi: 10.1037/0022-3514.91.1.63, PMID: 16834480

[ref123] Guinee L. N. Payne K. B. (1988). Rhyme-like repetitions in songs of humpback whales. Ethology 79, 295–306. doi: 10.1111/j.1439-0310.1988.tb00718.x

[ref124] Gunawardane K. G. Custance D. M. Piffer D. (2011). Evidence of sexual selection for evening orientation in human males: a cross cultural study in Italy and Sri Lanka. Interdiscip. Biol. Central 3, 13–17. doi: 10.4051/ibc.2011.3.4.0013

[ref125] Hagen E. H. (2022). The biological roots of music and dance: extending the credible signaling hypothesis to predator deterrence. Hum. Nat., 33, 261–279. doi: 10.1007/s12110-022-09429-9 35986877

[ref126] Hagen E. H. Bryant G. A. (2003). Music and dance as a coalition signalling system. Hum. Nat. 14, 21–51. doi: 10.1007/s12110-003-1015-z, PMID: 26189987

[ref127] Hagen E. H. Hammerstein P. (2009). Did Neanderthals and other early humans sing? Seeking the biological roots of music in the territorial advertisements of primates, lions, hyenas, and wolves. Mus. Sci. 13, 291–320. doi: 10.1177/1029864909013002131

[ref128] Haimoff E. H. (1986). Convergence in the duetting of monogamous Old World primates. J. Hum. Evol. 15, 51–59. doi: 10.1016/S0047-2484(86)80065-3

[ref129] Hallam S. (2006). Musicality Oxford University Press.

[ref130] Hallam S. (2010). The power of music: its impact on the intellectual, social and personal development of children and young people. Int. J. Music. Educ. 28, 269–289. doi: 10.1177/0255761410370658

[ref131] Hallett E. Y. Marean C. W. Steele T. E. Álvarez-Fernández E. Jacobs Z. Cerasoni J. N.. (2021). A worked bone assemblage from 120,000-90,000 year old deposits at Contrebandiers cave, Atlantic Coast, Morocco. iScience 24:102988. doi: 10.1016/j.isci.2021.102988, PMID: 34622180 PMC8478944

[ref132] Hargreaves D. J. North A. C. (1999). The functions of music in everyday life: redefining the social in music psychology. Psychol. Music 27, 71–83. doi: 10.1177/0305735699271007

[ref133] Haselton M. G. Nettle D. (2006). The paranoid optimist: an integrative evolutionary model of cognitive biases. Personal. Soc. Psychol. Rev. 10, 47–66. doi: 10.1207/s15327957pspr1001_3, PMID: 16430328

[ref134] Hattori Y. Tomonaga M. Matsuzawa T. (2013). Spontaneous synchronized tapping to an auditory rhythm in a chimpanzee. Sci. Rep. 3:1566. doi: 10.1038/srep01566 23535698 PMC3610097

[ref135] Hau M. Dominoni D. Casagrande S. Buck C. L. Wagner G. Hazlerigg D.. (2017). Timing as a sexually selected trait: the right mate at the right moment. Philos. Trans. R. Soc. B 372:20160249. doi: 10.1098/rstb.2016.0249, PMID: 28993493 PMC5647276

[ref136] Hauser M. D. McDermott J. (2003). The evolution of the music faculty: a comparative perspective. Nat. Neurosci. 6, 663–668. doi: 10.1038/nn1080, PMID: 12830156

[ref137] Heggli O. A. Stupacher J. Vuust P. (2021). Diurnal fluctuations in musical preference. R. Soc. Open Sci. 8:210885. doi: 10.1098/rsos.210885, PMID: 34804568 PMC8580447

[ref138] Hernandez-Aguilar R. A. Moore J. I. M. Stanford C. B. (2013). Chimpanzee nesting patterns in savanna habitat: environmental influences and preferences. Am. J. Primatol. 75, 979–994. doi: 10.1002/ajp.22163, PMID: 23653164

[ref139] Herzog N. M. Parker C. Keefe E. Hawkes K. (2020). Fire’s impact on threat detection and risk perception among vervet monkeys: implications for hominin evolution. J. Hum. Evol. 145:102836. doi: 10.1016/j.jhevol.2020.102836, PMID: 32619883

[ref140] Hirsh J. B. Galinsky A. D. Zhong C. B. (2011). Drunk, powerful, and in the dark: how general processes of disinhibition produce both prosocial and antisocial behavior. Pers. Psychol. Sci. 6, 415–427. doi: 10.1177/1745691611416992, PMID: 26168194

[ref141] Ho Y. Cheung M. Chan A. S. (2003). Music training improves verbal but not visual memory: crosssectional and longitudinal explorations in children. Neuropsychology 17, 439–450. doi: 10.1037/0894-4105.17.3.439, PMID: 12959510

[ref142] HobbsD. R.GallupG. G.Jr. (2011). Songs as a medium for embedded reproductive messages. Evol. Psychol. 9, 390–416. doi: 10.1177/147470491100900309, PMID: 22947982

[ref143] Honing H. (2018). The origins of musicality. Cambridge, MA: MIT Press.

[ref144] Honing H. Ploeger A. (2012). Cognition and the evolution of music: pitfalls and prospects. Top. Cogn. Sci. 4, 513–524. doi: 10.1111/j.1756-8765.2012.01210.x, PMID: 22760967

[ref145] Honing H. ten Cate C. Peretz I. Trehub S. E. (2015a). Without it no music: cognition, biology and evolution of musicality. Philos. Trans. R. Soc. Lond. Ser. B Biol. Sci. 370:88. doi: 10.1098/rstb.2014.0088 PMC4321129 25646511

[ref146] Honing H. ten Cate C. Peretz I. Trehub S. E. (2015b). Biology, cognition and origins of musicality [special issue]. Philos. Trans. R. Soc. Lond. Ser. B Biol. Sci. 370:88 10.1098/rstb.2014.0088 PMC4321129 25646511

[ref147] Honnebier M. B. O. M. Nathanielsz P. W. (1994). Primate parturition and the role of the maternal circadian system. Eur. J. Obstet. Gynecol. Reprod. Biol. 55, 193–203. doi: 10.1016/0028-2243(94)90038-8, PMID: 7958165

[ref148] Hooper P. L. Miller G. F. (2008). Mutual mate choice can drive costly signaling even under perfect monogamy. Adapt. Behav. 16, 53–70. doi: 10.1177/1059712307087283

[ref9007] Horwitz T. B. Balbona J. V. Paulich K. N. Keller M. C. (2023). Evidence of correlations between human partners based on systematic reviews and meta-analyses of 22 traits and UK Biobank analysis of 133 traits. Nat. Hum. Behav, 7, 1568–1583. doi: 10.1038/s41562-023-01672-z, PMID: 37653148 PMC10967253

[ref149] Hublin J. J. Ben-Ncer A. Bailey S. E. Freidline S. E. Neubauer S. Skinner M. M.. (2017). New fossils from Jebel Irhoud, Morocco and the pan-African origin of Homo sapiens. Nature 546, 289–292. doi: 10.1038/nature22336, PMID: 28593953

[ref150] Hugill N. Fink B. Neave N. Besson A. Bunse L. (2011). Women’s perception of men’s sensation seeking propensity from their dance movements. Personal. Individ. Differ. 51, 483–487. doi: 10.1016/j.paid.2011.05.002

[ref151] Huron D. (2001). Is music an evolutionary adaptation? Ann. N. Y. Acad. Sci. 930, 43–61. doi: 10.1111/j.1749-6632.2001.tb05724.x 11458859

[ref152] IJzerman H. Coan J. A. Wagemans F. M. A. Missler M. A. Beest I. v. Lindenberg S.. (2015). A theory of social thermoregulation in human primates. Front. Psychol. 6:464. doi: 10.3389/fpsyg.2015.00464, PMID: 25954223 PMC4404741

[ref153] Isbell L. A. Bidner L. R. (2016). Vervet monkeys (Chlorocebus pygerythrus) alarm calls to leopards (Panthera pardus) function as a predator deterrent. Behaviour 153, 591–606. doi: 10.1163/1568539X-00003365

[ref154] Isbell L. A. Bidner L. R. Van Cleave E. K. Matsumoto-Oda A. Crofoot M. C. (2018). GPS-identified vulnerabilities of savannah-woodland primates to leopard predation and their implications for early hominins. J. Hum. Evol. 118, 1–13. doi: 10.1016/j.jhevol.2018.02.003, PMID: 29606199

[ref155] Ismail M. J. Anuar A. F. Kamis M. S. (2020). Divergent thinking in musically gifted practices: a review. Quan. J. Soc. Sci. Hum. 1, 13–26. doi: 10.55197/qjssh.v1i5.22

[ref156] Ivarsdotter A. (2004). “And the cattle follow her, for they know her voice …on communication between women and cattle in Scandinavian pastures” in Pecus. Man and animal in antiquity. ed. Frizell B. S. (Rome: The Swedish Institute in Rome), 146–149.

[ref157] Jackendoff R. Lerdahl F. (2006). The capacity for music: what is it, and what's special about it? Cognition 100, 33–72. doi: 10.1016/j.cognition.2005.11.005, PMID: 16384553

[ref158] Jakobsen L. Christensen-Dalsgaard J. Juhl P. M. Elemans C. P. H. (2021). How loud can you go? Physical and physiological constraints to producing high sound pressures in animal vocalizations. Front. Ecol. Evol. 9:657254. doi: 10.3389/fevo.2021.657254

[ref159] Jakubowski K. Eerola T. Tillmann B. Perrin F. Heine L. (2020). A cross-sectional study of reminiscence bumps for music-related memories in adulthood. Music. Sci. 3:2059204320965058. doi: 10.1177/2059204320965058

[ref160] Jankowski K. S. Díaz-Morales J. F. Randler C. (2014). Chronotype, gender and time for sex. Chronobiol. Int. 31, 911–916. doi: 10.3109/07420528.2014.925470, PMID: 24927370

[ref161] Jarraya S. Jarraya M. (2019). The effects of music and the time-of-day on cognitive abilities of tennis player. J. Sport Exerc. Psychol. 17, 185–196. doi: 10.1080/1612197X.2017.1292299

[ref162] Jenkinson R. Bowring A. Dietze P. Hellard M. Lim M. S. (2014). Young risk takers: alcohol, illicit drugs and sexual practices among a sample of music festival attendees. J. Sex. Transm. Dis. 2014:357239. doi: 10.1155/2014/357239, PMID: 26316974 PMC4437411

[ref163] Jocz P. Stolarski M. Jankowski K. S. (2018). Similarity in chronotype and preferred time for sex and its role in relationship quality and sexual satisfaction. Front. Psychol. 9:443. doi: 10.3389/fpsyg.2018.00443 29670559 PMC5893780

[ref164] Jonason P. K. Foster J. D. McCain J. Campbell W. K. (2015). Where birds flock to get together: the who, what, where, and why of mate searching. Pers. Individ. Dif. 80, 76–84. doi: 10.1016/j.paid.2015.02.018

[ref165] Jonason P. K. Jones A. Lyons M. (2013). Creatures of the night: chronotypes and the dark triad traits. Personal. Individ. Differ. 55, 538–541. doi: 10.1016/j.paid.2013.05.001

[ref166] Jones S. E. Lane J. M. Wood A. R. Weedon M. N. (2019). Genome-wide association analyses of chronotype in 697,828 individuals provides insights into circadian rhythms. Nat. Commun. 10:343. doi: 10.1038/s41467-018-08259-7 30696823 PMC6351539

[ref167] Jordania J. (2011). Sexual selection or natural selection? New look on the evolution of human morphology, behavior and art. Kadmos 3, 400–411.

[ref168] Kalinowski K. Kozłowska A. Malesza M. Danel D. P. (2021). Evolutionary origins of music. Classical and recent hypotheses. Przegląd Antropologiczny 84, 213–231. doi: 10.2478/anre-2021-0011

[ref169] Kasaeian A. Weidenauer C. Hautzinger M. Randler C. (2019). Reproductive success, relationship orientation and sexual behaviour in heterosexuals: relationship with chronotype, sleep and sex. Evol. Psychol. 17:1474704919859760. doi: 10.1177/1474704919859760, PMID: 31272215 PMC10480892

[ref170] Kaufman S. B. Kozbelt A. Silvia P. Kaufman J. C. Ramesh S. Feist G. J. (2016). Who finds bill gates sexy? Creative mate preferences as a function of cognitive ability, personality, and creative achievement. J. Creat. Behav. 50, 294–307. doi: 10.1002/jocb.78

[ref171] Kedar Y. Kedar G. Barkai R. (2022). The influence of smoke density on hearth location and activity areas at lower Paleolithic lazaret cave, France. Sci. Rep. 12, 1–14. doi: 10.1038/s41598-022-05517-z 35087107 PMC8795116

[ref172] Keller P. E. König R. Novembre G. (2017). Simultaneous cooperation and competition in the evolution of musical behavior: sex-related modulations of the singer's formant in human chorusing. Front. Psychol. 8:1559. doi: 10.3389/fpsyg.2017.01559 28959222 PMC5603663

[ref173] Kennair L. E. O. Wade T. J. Tallaksen M. T. Grøntvedt T. V. Kessler A. M. Burch R. L.. (2022). Perceived effectiveness of flirtation tactics: the effects of sex, mating context and individual differences in US and Norwegian samples. Evol. Psychol. 20:1088011. doi: 10.1177/14747049221088011, PMID: 35331044 PMC10355297

[ref174] Kenny D. T. Asher A. (2016). Life expectancy and cause of death in popular musicians: is the popular musician lifestyle the road to ruin? Med. Probl. Perform. Art. 31, 37–44. doi: 10.21091/mppa.2016.1007, PMID: 26966963

[ref175] Killin A. (2017). Plio-pleistocene foundations of hominin musicality: coevolution of cogition, sociality, and music. Biol. Theor. 12, 222–235. doi: 10.1007/s13752-017-0274-6

[ref176] Killin A. (2018). The origins of music: evidence, theory, and prospects. Music. Sci. 1, 1–23. doi: 10.1177/2059204317751971

[ref177] Kirschner S. Tomasello M. (2010). Joint music making promotes prosocial behaviour in 4-year-old children. Evol. Hum. Behav. 31, 354–364. doi: 10.1016/j.evolhumbehav.2010.04.004

[ref178] Kleinman K. (2015). Darwin and Spencer on the origin of music: is music the food of love? Prog. Brain Res. 217, 3–15. doi: 10.1016/bs.pbr.2014.11.018 25725907

[ref179] Klinge C. Röder B. Büchel C. (2010). Increased amygdala activation to emotional auditory stimuli in the blind. Brain 133, 1729–1736. doi: 10.1093/brain/awq102, PMID: 20453040

[ref180] Klopfer P. H. Rubenstein D. I. (1977). The concept privacy and its biological basis. J. Soc. Issues 33, 52–65. doi: 10.1111/j.1540-4560.1977.tb01882.x, PMID: 35416786

[ref181] Knobloch S. Zillmann D. (2003). Appeal of love themes in popular music. Psychol. Rep. 93, 653–658. doi: 10.2466/pr0.2003.93.3.653, PMID: 14723423

[ref182] Kret M. E. Prochazkova E. Sterck E. H. Clay Z. (2020). Emotional expressions in human and non-human great apes. Neurosci. Biobehav. Rev. 115, 378–395. doi: 10.1016/j.neubiorev.2020.01.027, PMID: 31991191

[ref183] Kubik G. (2005). The African matrix in jazz harmonic practices. Black Music. Res. J. 25, 167–222.

[ref184] Lacroux C. Robira B. Kane-Maguire N. Guma N. Krief S. (2022). Between forest and croplands: nocturnal behavior in wild chimpanzees of Sebitoli, Kibale National Park Uganda. PloS One 17:E0268132. doi: 10.1371/journal.pone.0268132 35522693 PMC9075648

[ref185] Laeng B. Eidet L. M. Sulutvedt U. Panksepp J. (2016). Music chills: the eye pupil as a mirror to music’s soul. Conscious. Cogn. 44, 161–178. doi: 10.1016/j.concog.2016.07.009, PMID: 27500655

[ref186] Langergraber K. E. Prüfer K. Rowney C. Boesch C. Crockford C. Fawcett K.. (2012). Generation times in wild chimpanzees and gorillas suggest earlier divergence times in great ape and human evolution. Proc. Natl. Acad. Sci. U. S. A. 109, 15716–15721. doi: 10.1073/pnas.1211740109, PMID: 22891323 PMC3465451

[ref187] Large E. W. Gray P. (2015). Spontaneous tempo and entrainment in a bonobo (*Pan paniscus*). J. Comp. Psychol. 129, 317–328. doi: 10.1037/com0000011, PMID: 26147705

[ref188] Larom D. Garstang M. Payne K. Raspet R. Lindeque M. (1997). The influence of surface atmospheric conditions on the range and area reached by animal vocalizations. J. Exp. Biol. 200, 421–431. doi: 10.1242/jeb.200.3.421, PMID: 9057305

[ref189] Lauer G. (2022). Language, childhood, and fire: how we learned to love sharing stories. Front. Psychol. 12:787203. doi: 10.3389/fpsyg.2021.787203, PMID: 35153908 PMC8828489

[ref190] Launay J. Tarr B. Dunbar R. (2016). Synchrony as an adaptive mechanism for large-scale human social bonding. Ethology 122, 779–789. doi: 10.1111/eth.12528

[ref191] Leanderson R. Sundberg J. Von Euler C. (1987). Breathing muscle activity and subglottal pressure dynamics in singing and speech. J. Voice 1, 258–261. doi: 10.1016/S0892-1997(87)80009-7

[ref192] Lee-Thorp J. Thackeray J. F. van der Merwe N. (2000). The hunters and the hunted revisited. J. Hum. Evol. 39, 565–576. doi: 10.1006/jhev.2000.0436 11102267

[ref193] Lehman C. Welker L. Schiefenhövel W. (2009). Towards an ethology of song: a categorization of musical behaviour. Music. Sci. 13, 321–338. doi: 10.1177/1029864909013002141

[ref194] Lehmiller J. J. (2018). Tell me what you want: The science of sexual desire and how it can help you improve your sex life. New York, NY: Da Capo Press.

[ref195] Leongómez J. D. Havlíček J. Roberts S. C. (2022). Musicality in human vocal communication: an evolutionary perspective. Philos. Trans. R. Soc. Lond. Ser. B Biol. Sci. 377:391. doi: 10.1098/rstb.2020.0391 PMC8591388 34775823

[ref196] Levitin D. J. (2008). The world in six songs: How the musical brain created human nature. London, UK: Atlantic.

[ref197] Levitin D. J. (2021). Knowledge songs as an evolutionary adaptation to facilitate information transmission through music. Behav. Brain Sci. 44:E105. doi: 10.1017/S0140525X20001090 34588034

[ref198] Levos J. Zacchilli T. L. (2015). Nyctophobia: from imagined to realistic fears of the dark. Psi Chi J. Psychol. Res. 20, 102–110. doi: 10.24839/2164-8204.JN20.2.102

[ref199] Lewis D. M. G. Conroy-Beam D. Asao K. Buss D. M. (2017). Evolutionary psychology: a how-to guide. Am. Psychol. 72, 353–373. doi: 10.1037/a0040409, PMID: 28481582

[ref200] Li H. (2022). Normal lark, deviant owl: the relationship between chronotype and compliance with COVID-19 mitigation measures. Chronobiol. Int. 39, 1524–1532. doi: 10.1080/07420528.2022.2123276, PMID: 36221303

[ref201] Li Y. Ma W. Kang Q. Qiao L. Tang D. Qiu J.. (2015). Night or darkness, which intensifies the feeling of fear? J. Psychophysiol. 97, 46–57. doi: 10.1016/j.ijpsycho.2015.04.021 25957698

[ref202] Lindblom B. Sundberg J. (2014). “The human voice in speech and singing” in Springer handbook of acoustics. ed. Rossing T. D. (Berlin/Heidelberg: Springer), 703–746.

[ref203] Linnemann A. Strahler J. Nater U. M. (2016). The stress-reducing effect of music listening varies depending on the social context. Psychoneuroendocrinology 72, 97–105. doi: 10.1016/j.psyneuen.2016.06.003, PMID: 27393906

[ref204] Little P. Zuckerman M. (1986). Sensation seeking and music preferences. Pers. Individ. Differ. 7, 575–577. doi: 10.1016/0191-8869(86)90136-4, PMID: 34484021

[ref205] Loersch C. Arbuckle N. L. (2013). Unraveling the mystery of music: music as an evolved group process. J. Pers. Soc. Psychol. 105, 777–798. doi: 10.1037/a0033691, PMID: 23895270

[ref206] Lombardo M. P. Deaner R. O. (2018). Born to throw: the ecological causes that shaped the evolution of throwing in humans. Q. Rev. Biol. 93, 1–16. doi: 10.1086/696721

[ref207] Lorenz K. (1981). The foundations of ethology. New York: Springer Science+ Business Media.

[ref208] Loveday C. Woy A. Conway M. A. (2020). The self-defining period in autobiographical memory: evidence from a long-running radio show. Q. J. Exp. Physiol. 73, 1969–1976. doi: 10.1177/1747021820940300 PMC7583440 32564690

[ref209] Macfarlane A. Dattani N. Gibson R. Harper G. Martin P. Scanlon M.. (2019). Births and their outcomes by time, day and year: a retrospective birth cohort data linkage study. Health Serv. Deliv. Res. 7:hsdr07180. doi: 10.3310/hsdr07180 31141328

[ref210] Madison G. Schiölde G. (2017). Repeated listening increases the liking for music regardless of its complexity: implications for the appreciation and aesthetics of music. Front. Neurosci. 11:147. doi: 10.3389/fnins.2017.00147, PMID: 28408864 PMC5374342

[ref211] MaestripieriD. (2014). Night owl women are similar to men in their relationship orientation, risk-taking propensities, and cortisol levels: implications for the adaptive significance and evolution of eveningness. Evol. Psychol. 12, 130–147. doi: 10.1177/147470491401200111, PMID: 24566433

[ref212] Mafra A. L. Varella M. A. C. Defelipe R. P. Anchieta N. M. de Almeida C. A. G. Valentova J. V. (2020). Makeup usage in women as a tactic to attract mates and compete with rivals. Pers. Individ. Differ. 163:110042. doi: 10.1016/j.paid.2020.110042

[ref213] Mannion H. Hendrie C. Godfrey G. (2009). Evidence to suggest that nightclubs function as human sexual display grounds. Behaviour 146, 1331–1348. doi: 10.1163/156853909X427704

[ref214] Marin M. M. Rathgeber I. (2022). Darwin’s sexual selection hypothesis revisited: musicality increases sexual attraction in both sexes. Front. Psychol. 13:971988. doi: 10.3389/fpsyg.2022.971988, PMID: 36092107 PMC9453251

[ref215] Marin M. M. Schober R. Gingras B. Leder H. (2017). Misattribution of musical arousal increases sexual attraction towards opposite-sex faces in females. PLoS One 12:e0183531. doi: 10.1371/journal.pone.0183531, PMID: 28892486 PMC5593195

[ref216] Martin P. Cortina-Borja M. Newburn M. Harper G. Gibson R. Dodwell M.. (2018). Timing of singleton births by onset of labour and mode of birth in NHS maternity units in England, 2005–2014: a study of linked birth registration, birth notification, and hospital episode data. PLoS One 13:E0198183. doi: 10.1371/journal.pone.0198183 29902220 PMC6002087

[ref9008] Marvel-Coen J. Scrivner C. Maestripieri D. (2018). “Morningness–eveningness and sociosexuality from a life history perspective”, in The SAGE Handbook of Personality and Individual Differences. Eds. V. Zeigler-Hill and T. Shackelford (Thousand Oaks, CA: Sage).

[ref217] Masataka N. (2009). The evolution of music: theories, definitions and the nature of the evidence. The origins of language and the evolution of music: a comparative perspective. Phys Life Rev 6, 11–22. doi: 10.1016/j.plrev.2008.08.003, PMID: 22537940

[ref218] Matchock R. L. (2018). Evening chronotype is associated with a more unrestricted sociosexuality in men and women. Pers. Indiv. Dif. 135, 56–59. doi: 10.1016/j.paid.2018.06.054

[ref219] May J. L. Hamilton P. A. (1980). Effects of musically evoked affect on women's interpersonal attraction toward and perceptual judgments of physical attractiveness of men. Motiv. Emot. 4, 217–228. doi: 10.1007/BF00995420

[ref220] McCaffrey T. Cheung P. S. Barry M. Punch P. Dore L. (2020). The role and outcomes of music listening for women in childbirth: an integrative review. Midwifery 83:102627. doi: 10.1016/j.midw.2020.102627, PMID: 31951943

[ref221] McFarland R. Henzi S. P. Fuller A. Hetem R. S. Young C. Barrett L. (2022). The thermal consequences of primate birth hour and its evolutionary implications. Biol. Lett. 18:20210574. doi: 10.1098/rsbl.2021.0574 35078330 PMC8790368

[ref222] McGhee G. R. (2011). Convergent evolution: Limited forms most beautiful. Cambridge, MA: MIT press.

[ref223] McManus I. C. Furnham A. (2006). Aesthetic activities and aesthetic attitudes: influences of education, background and personality on interest and involvement in the arts. Br. J. Psychol. 97, 555–587. doi: 10.1348/000712606X101088, PMID: 17018189

[ref224] Mehr S. A. Krasnow M. M. Bryant G. A. Hagen E. H. (2021). Origins of music in credible signaling. Behav. Brain Sci. 44:E60. doi: 10.1017/S0140525X20000345 PMC7907251 32843107

[ref225] Mehr S. A. Singh M. Knox D. Ketter D. M. Pickens-Jones D. Atwood S.. (2019). Universality and diversity in human song. Science 366:17. doi: 10.1126/science.aax0868 PMC7001657 31753969

[ref226] Mehr S. A. Singh M. York H. Glowacki L. Krasnow M. M. (2018). Form and function in human song. Curr. Biol. 28, 356.e5–368.e5. doi: 10.1016/j.cub.2017.12.042 29395919 PMC5805477

[ref227] Menninghaus W. (2019). Aesthetics after Darwin: the multiple origins and functions of art. Boston: Academic Studies Press.

[ref228] Merker B. (1999). Synchronous chorusing and the origins of music. Music. Sci. 3, 59–73. doi: 10.1177/10298649000030S105, PMID: 24429686

[ref229] Merker B. (2021). Music, bonding, and human evolution: a critique. Behav. Brain Sci. 44:E83. doi: 10.1017/S0140525X20001429 34588063

[ref230] Merker B. Morley I. Zuidema W. (2015). Five fundamental constraints on theories of the origin of music. Philos. Trans. R. Soc. B 370:20140095. doi: 10.1098/rstb.2014.0095, PMID: 25646518 PMC4321136

[ref231] Merriam A. P. (1964). The anthropology of music. Chicago: Northwestern University Press.

[ref232] Micoogullari U. Kisa E. Celik O. Erbay O. F. Koc E. Gok B. (2021). New behavior therapy in the treatment of acquired premature ejaculation: a comparative study of listening to music. Arch. Esp. Urol. 74, 519–525. PMID: 34080572

[ref233] Mikula P. Valcu M. Brumm H. Bulla M. Forstmeier W. Petrusková T.. (2021). A global analysis of song frequency in passerines provides no support for the acoustic adaptation hypothesis but suggests a role for sexual selection. Ecol. Lett. 24, 477–486. doi: 10.1111/ele.13662, PMID: 33314573

[ref234] Miles S. A. Miranda R. A. Ullman M. T. (2016). Sex differences in music: a female advantage at recognizing familiar melodies. Front. Psychol. 7:278. doi: 10.3389/fpsyg.2016.00278, PMID: 26973574 PMC4771742

[ref235] Millar B. M. Rendina H. J. Starks T. J. Grov C. Parsons J. T. (2019). The role of chronotype, circadian misalignment, and tiredness in the substance use behaviors of gay and bisexual men. Psychol. Sex. Orientat. Gend. Divers. 6, 96–106. doi: 10.1037/sgd0000311, PMID: 30906800 PMC6426147

[ref236] Miller G. (2000). “Evolution of human music through sexual selection” in The origins of music. eds. Wallin N. L. Merker B. Brown S. (Cambridge: The MIT Press), 329–360.

[ref237] Miller G. Tybur J. M. Jordan B. D. (2007). Ovulatory cycle effects on tip earnings by lap dancers: economic evidence for human estrus? Evol. Hum. Behav. 28, 375–381. doi: 10.1016/j.evolhumbehav.2007.06.002

[ref238] Mindell J. A. Williamson A. A. (2018). Benefits of a bedtime routine in young children: sleep, development, and beyond. Sleep Med. Rev. 40, 93–108. doi: 10.1016/j.smrv.2017.10.007, PMID: 29195725 PMC6587181

[ref239] Mingle M. E. Eppley T. M. Campbell M. W. Hall K. Horner V. De Waal F. (2014). Chimpanzees prefer African and Indian music over silence. J. Exp. Psychol. Anim. Learn. Cogn. 40, 502–505. doi: 10.1037/xan0000032, PMID: 25546107 PMC4461656

[ref240] Mitani J. C. Stuht J. (1998). The evolution of nonhuman primate loud calls: acoustic adaptation for long-distance transmission. Primates 39, 171–182. doi: 10.1007/BF02557729

[ref241] Mithen S. (2005). The singing Neanderthals: The origins of music, language, mind and body. London: Weidenfeld and Nicolson.

[ref242] Mithen S. (2009). The music instinct: the evolutionary basis of musicality. Ann. N. Y. Acad. Sci. 1169, 3–12. doi: 10.1111/j.1749-6632.2009.04590.x 19673750

[ref243] Mithen S. (2019). Mesolithic fireplaces and the enculturation of early holocene landscapes in Britain, with a case study from Western Scotland. P. Prehist. Soc. 85, 131–159. doi: 10.1017/ppr.2019.6

[ref244] Mobbs D. (2018). The ethological deconstruction of fear(s). Curr. Opin. Behav. Sci. 24, 32–37. doi: 10.1016/j.cobeha.2018.02.008, PMID: 31467943 PMC6715320

[ref245] Moraes Y. L. Valentova J. V. Varella M. A. C. (2022). The evolution of playfulness, play and play-like phenomena in relation to sexual selection. Front. Psychol. 13:925842. doi: 10.3389/fpsyg.2022.925842, PMID: 35756316 PMC9226980

[ref246] Moreno S. Bialystok E. Barac R. Schellenberg E. G. Cepeda N. J. Chau T. (2011). Short-term music training enhances verbal intelligence and executive function. Psychol. Sci. 22, 1425–1433. doi: 10.1177/0956797611416999, PMID: 21969312 PMC3449320

[ref247] Morley I. (2013). The prehistory of music: Human evolution, archaeology, and the origins of musicality. Oxford: Oxford University Press.

[ref248] Mosing M. A. Madison G. Pedersen N. L. Kuja-Halkola R. Ullén F. (2014). Practice does not make perfect. Psychol. Sci. 25, 1795–1803. doi: 10.1177/0956797614541990, PMID: 25079217

[ref249] Mosing M. A. Verweij K. J. Madison G. Pedersen N. L. Zietsch B. P. Ullén F. (2015). Did sexual selection shape human music? Testing predictions from the sexual selection hypothesis of music evolution using a large genetically informative sample of over 10,000 twins. Evol. Hum. Behav. 36, 359–366. doi: 10.1016/j.evolhumbehav.2015.02.004

[ref250] Musharbash Y. (2013). Night, sight, and feeling safe: an exploration of aspects of Warlpiri and Western sleep. Aust. J. Anthropol. 24, 48–63. doi: 10.1111/taja.12021

[ref251] Needham A. Wisher I. Langley A. Amy M. Little A. (2022). Art by firelight? Using experimental and digital techniques to explore Magdalenian engraved plaquette use at Montastruc (France). PLoS One 17:E0266146. doi: 10.1371/journal.pone.0266146 35442964 PMC9020732

[ref252] Nesse R. M. (2001). The smoke detector principle: natural selection and the regulation of defensive responses. Ann. N. Y. Acad. Sci. 935, 75–85. doi: 10.1111/j.1749-6632.2001.tb03472.x, PMID: 11411177

[ref253] Nettle D. Scott-Phillips T. (2023). “Is a non-evolutionary psychology possible?” in Evolutionary thinking across disciplines: problems and perspectives in generalized Darwinism. eds. Crest A. Valkovic M. Ariew A. Desmond H. Huneman P. Reydon T. (New York, NY: Springer), 27–55.

[ref254] Niarchou M. Gustavson D. E. Sathirapongsasuti J. F. Anglada-Tort M. Eising E. Bell E.. (2022). Genome-wide association study of musical beat synchronization demonstrates high polygenicity. Nat. Hum. Behav. 6, 1292–1309. doi: 10.1038/s41562-022-01359-x, PMID: 35710621 PMC9489530

[ref255] Nikolsky A. Perlovsky L. (2020). The evolution of music [special issue]. Front. Psychol. 11:595517. doi: 10.3389/fpsyg.2020.595517, PMID: 33192939 PMC7655650

[ref256] North A. Hargreaves D. (2008). The social and applied psychology of music. New York: Oxford University Press.

[ref257] Nowack K. Van Der Meer E. (2013). Are larks future-oriented and owls present-oriented? Age-and sex-related shifts in chronotype–time perspective associations. Chronobiol. Int. 30, 1240–1250. doi: 10.3109/07420528.2013.815197, PMID: 24073885

[ref258] Nowell A. (2018). “Upper Paleolithic soundscapes and the emotional resonance of nighttime” in Archaeology of the night: Life after dark in the ancient world. eds. Gonlin N. Nowell A. (Boulder, CO: University Press of Colorado), 27–44.

[ref259] Nunn C. L. Samson D. R. (2018). Sleep in a comparative context: investigating how human sleep differs from sleep in other primates. Am. J. Phys. Anthropol. 166, 601–612. doi: 10.1002/ajpa.23427, PMID: 29446072

[ref260] Nunn C. L. Samson D. R. Krystal A. D. (2016). Shining evolutionary light on human sleep and sleep disorders. Evol. Med. Public Health 2016, 227–243. doi: 10.1093/emph/eow018, PMID: 27470330 PMC4972941

[ref261] O’Connell J. F. Hawkes K. Jones N. B. (1999). Grandmothering and the evolution of Homo erectus. J. Hum. Evol. 36, 461–485. doi: 10.1006/jhev.1998.0285, PMID: 10222165

[ref262] Oh C. M. Kim H. Y. Na H. Cho K. H. Chu M. K. (2019). The effect of anxiety and depression on sleep quality of individuals with high risk for insomnia: a population-based study. Front. Neurol. 10:849. doi: 10.3389/fneur.2019.00849, PMID: 31456736 PMC6700255

[ref263] Oikkonen J. Onkamo P. Järvelä I. Kanduri C. (2016). Convergent evidence for the molecular basis of musical traits. Sci. Rep. 6:39707. doi: 10.1038/srep39707 28004803 PMC5177873

[ref264] Ottoni G. L. Antoniolli E. Lara D. R. (2012). Circadian preference is associated with emotional and affective temperaments. Chronobiol. Int. 29, 786–793. doi: 10.3109/07420528.2012.679329, PMID: 22734579

[ref265] Packer C. Swanson A. Ikanda D. Kushnir H. (2011). Fear of darkness, the full moon and the nocturnal ecology of African lions. PLoS One 6:E22285. doi: 10.1371/journal.pone.0022285 21799812 PMC3140494

[ref266] Park M. Thom J. Mennicken S. Cramer H. Macy M. (2019). Global music streaming data reveal diurnal and seasonal patterns of affective preference. Nat. Hum. Behav. 3, 230–236. doi: 10.1038/s41562-018-0508-z, PMID: 30953008

[ref267] Parker C. H. Keefe E. R. Herzog N. M. O’connell J. F. Hawkes K. (2016). The pyrophilic primate hypothesis. Evol. Anthropol. 25, 54–63. doi: 10.1002/evan.21475, PMID: 27061034

[ref268] Patel A. D. (2018). “Music as a transformative technology of the mind: an update” in The origins of musicality. ed. Honing H. (Cambridge, MA: MIT Press), 113–126.

[ref269] Pearce E. Launay J. van Duijn M. Rotkirch A. David-Barrett T. Dunbar R. I. M. (2016). Singing together or apart: the effect of competitive and cooperative singing on social bonding within and between sub-groups of a university fraternity. Psychol. Music 44, 1255–1273. doi: 10.1177/0305735616636208, PMID: 27777494 PMC5074360

[ref270] Penke L. Asendorpf J. B. (2008). Beyond global sociosexual orientations: a more differentiated look at sociosexuality and its effects on courtship and romantic relationships. J. Pers. Soc. Psychol. 95:1113. doi: 10.1037/0022-3514.95.5.1113, PMID: 18954197

[ref271] Peretz I. (2006). The nature of music from a biological perspective. Cognition 100, 1–32. doi: 10.1016/j.cognition.2005.11.004 16487953

[ref272] Peretz I. Coltheart M. (2003). Modularity of music processing. Nat. Neurosci. 6, 688–691. doi: 10.1038/nn1083, PMID: 12830160

[ref273] Piasecki T. M. Jahng S. Wood P. K. Robertson B. M. Epler A. J. Cronk N. J.. (2011). The subjective effects of alcohol–tobacco co-use: an ecological momentary assessment investigation. J. Abnorm. Psychol. 120, 557–571. doi: 10.1037/a0023033, PMID: 21443289 PMC3128190

[ref274] Piel A. K. (2018). Temporal patterns of chimpanzee loud calls in the Issa Valley, Tanzania: evidence of nocturnal acoustic behavior in wild chimpanzees. Am. J. Phys. Anthropol. 166, 530–540. doi: 10.1002/ajpa.23609, PMID: 29989161

[ref275] Piffer D. (2010). Sleep patterns and sexual selection: an evolutionary approach. Mank. Q. 50:361. doi: 10.46469/mq.2010.50.4.6, PMID: 27454254

[ref276] Pinker S. (1997). How the mind works (Norton Pbk). New York: Norton.

[ref277] Pinker S. (2005). “Foreword” in The handbook of evolutionary psychology. ed. Buss D. M. (New Jersey: John Wiley & Sons), xi–xvi.

[ref278] Plomin R. DeFries J. C. Knopik V. S. Neiderhiser J. M. (2016). Top 10 replicated findings from behavioral genetics. Perspect. Psychol. Sci. 11, 3–23. doi: 10.1177/1745691615617439, PMID: 26817721 PMC4739500

[ref279] Polderman T. J. Benyamin B. de Leeuw C. A. Sullivan P. F. van Bochoven A. Visscher P. M.. (2015). Meta-analysis of the heritability of human traits based on fifty years of twin studies. Nat. Genet. 47, 702–709. doi: 10.1038/ng.3285, PMID: 25985137

[ref280] Ponzi D. Henry A. Kubicki K. Nickels N. Wilson M. C. Maestripieri D. (2015a). Morningness–eveningness and intrasexual competition in men. Pers. Individ. Dif. 76, 228–231. doi: 10.1016/j.paid.2014.12.023

[ref281] Ponzi D. Henry A. Kubicki K. Nickels N. Wilson M. C. Maestripieri D. (2015b). The slow and fast life histories of early birds and night owls: their future-or present-orientation accounts for their sexually monogamous or promiscuous tendencies. Evol. Hum. Behav. 36, 117–122. doi: 10.1016/j.evolhumbehav.2014.09.008

[ref282] Ponzi D. Wilson M. C. Maestripieri D. (2014). Eveningness is associated with higher risk-taking, independent of sex and personality. Psychol. Rep. 115, 932–947. doi: 10.2466/19.12.PR0.115c28z5, PMID: 25457099

[ref283] Porfírio J. C. C. Varella M. A. C. (2022). Testing the cognitive niche hypothesis with structural equation modeling: different dark traits predict an evening-chronotype in males and females. Curr. Psychol.:s12144-022-04111-w. doi: 10.1007/s12144-022-04111-w

[ref284] Power C. (1999). “‘Beauty magic’: the origins of art” in The evolution of culture. eds. Dunbar R. Knight C. Power C. (New Brunswick, NJ: Rutgers University Press), 92–112.

[ref285] Pozzi L. Voskamp M. Kappeler P. M. (2022). The effects of body size, activity, and phylogeny on primate sleeping ecology. Am. J. Biol. Anthropol. 179, 598–608. doi: 10.1002/ajpa.24640

[ref286] Preckel F. Lipnevich A. A. Schneider S. Roberts R. D. (2011). Chronotype, cognitive abilities, and academic achievement: a meta-analytic investigation. Learn. Individ. Diff. 21, 483–492. doi: 10.1016/j.lindif.2011.07.003

[ref287] Pruetz J. D. (2018). Nocturnal behavior by a diurnal ape, the West African chimpanzee (*Pan troglodytes verus*), in a savanna environment at Fongoli. Senegal. Am. J. Phys. Anthropol. 166, 541–548. doi: 10.1002/ajpa.23434, PMID: 29417991

[ref288] Putilov A. A. (2014). What were “owls” doing in our ancestral photoperiodic environment? Chronobiological account for the evolutionary advantage of nocturnal lifestyle. Biol. Rhythm. Res. 45, 759–787. doi: 10.1080/09291016.2014.913950

[ref289] Putilov A. A. (2021). Quo Vadis, Chronopsychology? Neurosci. Behav. Physiol. 51, 1244–1261. doi: 10.1007/s11055-021-01187-y

[ref290] Puts D. A. (2010). Beauty and the beast: mechanisms of sexual selection in humans. Evol. Hum. Behav. 31, 157–175. doi: 10.1016/j.evolhumbehav.2010.02.005

[ref291] Quenette P. Y. (1990). Functions of vigilance behaviour in mammals: a review. Acta Oecol. 11, 801–818.

[ref292] Randler C. (2016). Chronotype in children and adolescents. Somnologie 20, 166–171. doi: 10.1007/s11818-016-0073-5, PMID: 37697814

[ref293] Randler C. Kretz S. (2011). Assortative mating in morningness–eveningness. Int. J. Psychol. 46, 91–96. doi: 10.1080/00207594.2010.518237, PMID: 22044180

[ref294] Randler C. Schredl M. Göritz A. S. (2017). Chronotype, sleep behavior, and the big five personality factors. SAGE Open 7:728321. doi: 10.1177/2158244017728321

[ref295] Rawlings D. Barrantes i Vidal N. Furnham A. (2000). Personality and aesthetic preference in Spain and England: two studies relating sensation seeking and openness to experience to liking for paintings and music. Eur. J. Personal. 14, 553–576. doi: 10.1002/1099-0984(200011/12)14:6<553::AID-PER384>3.0.CO;2-H

[ref296] Reagh Z. M. Murray E. A. Yassa M. A. (2017). Repetition reveals ups and downs of hippocampal, thalamic, and neocortical engagement during mnemonic decisions. Hippocampus 27, 169–183. doi: 10.1002/hipo.22681, PMID: 27859884 PMC5858562

[ref297] Redmond E. M. (2016). Meeting with resistance: early Spanish encounters in the Americas, 1492–1524. Ethnohistory 63, 671–695. doi: 10.1215/00141801-3633264

[ref298] Refinetti R. (2005). Time for sex: nycthemeral distribution of human sexual behavior. J. Circadian Rhythms 3:4. doi: 10.1186/1740-3391-3-4 15790406 PMC1079926

[ref299] Reznikoff I. (2008). Sound resonance in prehistoric times: a study of Paleolithic painted caves and rocks. J. Acoust. Soc. Am. 123:3603. doi: 10.1121/1.2934773

[ref300] Roberts K. R. Dimsdale J. East P. Friedman L. (1998). Adolescent emotional response to music and its relationship to risk-taking behaviors. J. Adolesc. Health 23, 49–54. doi: 10.1016/S1054-139X(97)00267-X, PMID: 9648022

[ref301] Röder S. Carbon C.-C. Shackelford T. K. Pisanski K. Weege B. Fink B. (2016). Men’s visual attention and perceptions of women’s dance movements. Pers. Individ. Differ. 101, 1–3. doi: 10.1016/j.paid.2016.05.025

[ref302] Röder B. Rösler F. Neville H. J. (2001). Auditory memory in congenitally blind adults: a behavioral-electrophysiological investigation. Cogn. Brain Res. 11, 289–303. doi: 10.1016/S0926-6410(01)00002-7, PMID: 11275490

[ref303] Roebroeks W. Villa P. (2011). On the earliest evidence for habitual use of fire in Europe. Proc. Natl. Acad. Sci. U. S. A. 108, 5209–5214. doi: 10.1073/pnas.1018116108, PMID: 21402905 PMC3069174

[ref304] Rolland N. (2004). Was the emergence of home bases and domestic fire a punctuated event? A review of the middle Pleistocene record in Eurasia. Asian Perspect. 43, 248–280. doi: 10.1353/asi.2004.0027

[ref305] Román-Caballero R. Vadillo M. A. Trainor L. J. Lupiánez J. (2022). Please don't stop the music: a meta-analysis of the cognitive and academic benefits of instrumental musical training in childhood and adolescence. Educ. Res. Rev. 35:100436. doi: 10.1016/j.edurev.2022.100436

[ref306] Römer H. (2001). “Ecological constraints for sound communication: from grasshoppers to elephants” in Ecology of sensing. eds. Barth F. G. Schmid A. (Berlin: Springer), 59–78.

[ref307] Salmon C. (2018). Evolutionary perspectives on popular culture: state of the art. Evol. Stud. Imaginative Cult. 2, 47–66. doi: 10.26613/esic.2.2.92

[ref308] Samson D. R. (2021). The human sleep paradox: the unexpected sleeping habits of *Homo sapiens*. Annu. Rev. Anthropol. 50, 259–274. doi: 10.1146/annurev-anthro-010220-075523, PMID: 34803618

[ref309] Samson D. R. Crittenden A. N. Mabulla I. A. Mabulla A. Z. Nunn C. L. (2017). Chronotype variation drives night-time sentinel-like behaviour in hunter–gatherers. Proc. R. Soc. B Biol. Sci. 284:967. doi: 10.1098/rspb.2017.0967, PMID: 28701566 PMC5524507

[ref310] Samson S. Dellacherie D. Platel H. (2009). Emotional power of music in patients with memory disorders. Ann. N. Y. Acad. Sci. 1169, 245–255. doi: 10.1111/j.1749-6632.2009.04555.x 19673788

[ref311] Samson D. R. Hurst D. Shumaker R. W. (2014). Orangutan night-time long call behavior: sleep quality costs associated with vocalizations in captive Pongo. Adv. Zoo. 2014:101763. doi: 10.1155/2014/101763

[ref312] Samson D. R. Nunn C. L. (2015). Sleep intensity and the evolution of human cognition. Evol. Anthropol. 24, 225–237. doi: 10.1002/evan.21464, PMID: 26662946

[ref313] Savage P. E. Brown S. Sakai E. Currie T. E. (2015). Statistical universals reveal the structures and functions of human music. Proc. Natl. Acad. Sci. U. S. A. 112, 8987–8992. doi: 10.1073/pnas.1414495112, PMID: 26124105 PMC4517223

[ref314] Savage P. Loui P. Tarr B. Schachner A. Glowacki L. Mithen S.. (2021). Music as a coevolved system for social bonding. Behav. Brain Sci. 44:E59. doi: 10.1017/S0140525X20000333 32814608

[ref315] Savage P. E. Loui P. Tarr B. Schachner A. Glowacki L. Mithen S.. (2021). Toward inclusive theories of the evolution of musicality. Behav. Brain Sci. 44:E121. doi: 10.1017/S0140525X21000042 34588076 PMC13352484

[ref316] Schäfer T. Sedlmeier P. Städtler C. Huron D. (2013). The psychological functions of music listening. Front. Psychol. 4:511. doi: 10.3389/fpsyg.2013.00511, PMID: 23964257 PMC3741536

[ref317] Schellenberg E. G. (2004). Music lessons enhance IQ. Psychol. Sci. 15, 511–514. doi: 10.1111/j.0956-7976.2004.00711.x, PMID: 15270994

[ref318] Schellenberg E. G. (2011). Music lessons, emotional intelligence, and IQ. Music. Percept. 29, 185–194. doi: 10.1525/mp.2011.29.2.185, PMID: 22642351

[ref319] Schmitt D. P. Pilcher J. J. (2004). Evaluating evidence of psychological adaptation: how do we know one when we see one? Psychol. Sci. 15, 643–649. doi: 10.1111/j.0956-7976.2004.00734.x, PMID: 15447633

[ref320] Schruth D. M. Templeton C. N. Holman D. J. (2021). On reappearance and complexity in musical calling. PLoS One 16:E0218006. doi: 10.1371/journal.pone.0218006 34919558 PMC8683036

[ref321] Schulkind M. D. (2009). Is memory for music special? Ann. N. Y. Acad. Sci. 1169, 216–224. doi: 10.1111/j.1749-6632.2009.04546.x 19673785

[ref322] Scott R. V. Hosfield R. (2021). Fire in the round: a holistic approach to the lower Palaeolithic record. J. Archaeolog. Sci. Rep. 37:102938. doi: 10.1016/j.jasrep.2021.102938

[ref323] Searcy W. A. Andersson M. (1986). Sexual selection and the evolution of song. Annu. Rev. Ecol. Evol. Syst. 17, 507–533. doi: 10.1146/annurev.es.17.110186.002451, PMID: 37403502

[ref324] Sevincer G. M. Ince E. Taymur I. Konuk N. (2016). Night eating syndrome frequency in university students: association with impulsivity, depression, and anxiety. J. Clin. Psychopharmacol. 26, 238–247. doi: 10.5455/bcp.20160322093750

[ref325] Sheldon R. M. (2012). Ambush: Surprise attack in ancient Greek warfare. Philadelphia: Casemate Publishers.

[ref326] Sloboda J. A. O'Neill S. A. Ivaldi A. (2001). Functions of music in everyday life: an exploratory study using the experience sampling method. Music. Sci. 5, 9–32. doi: 10.1177/102986490100500102

[ref327] Smith D. Schlaepfer P. Major K. Dyble M. Page A. E. Thompson J.. (2017). Cooperation and the evolution of hunter-gatherer storytelling. Nat. Commun. 8:1853. doi: 10.1038/s41467-017-02036-8 29208949 PMC5717173

[ref328] Snowdon C. T. Zimmermann E. Altenmüller E. (2015). “Music evolution and neuroscience” in Progress in brain research. eds. Altenmüller E. Finger S. Boller F., vol. 217 (Amsterdam: Elsevier), 17–34. 10.1016/bs.pbr.2014.11.019 25725908

[ref329] Soma M. Garamszegi L. Z. (2011). Rethinking birdsong evolution: meta-analysis of the relationship between song complexity and reproductive success. Behav. Ecol. 22, 363–371. doi: 10.1093/beheco/arq219

[ref330] Sorokowska A. Oleszkiewicz A. Sorokowski P. (2018). A compensatory effect on mate selection? Importance of auditory, olfactory and tactile cues in partner choice among blind and sighted individuals. Arch. Sex. Behav. 47, 597–603. doi: 10.1007/s10508-018-1156-0, PMID: 29396613 PMC5834579

[ref331] Sotiropoulos M. G. Anagnostouli M. (2021). Genes, brain dynamics and art: the genetic underpinnings of creativity in dancing, musicality and visual arts. J. Integr. Neurosci. 20, 1095–1104. doi: 10.31083/j.jin2004110, PMID: 34997732

[ref332] Spencer H. (1875). “The origin and function of music” in Illustrations of universal progress: a series of discussions. ed. Spencer H. (New York, NY: D Appleton & Company), 210–238.

[ref333] Sprajcer M. Stewart D. Miller D. Lastella M. (2022). Sleep and sexual satisfaction in couples with matched and mismatched chronotypes: a dyadic cross-sectional study. Chronobiol. Int. 39, 1249–1255. doi: 10.1080/07420528.2022.2093213, PMID: 35762311

[ref334] Sterelny K. (2018). Religion re-explained. Relig. Brain Behav. 8, 406–425. doi: 10.1080/2153599X.2017.1323779

[ref335] Stewart-Williams S. Thomas A. G. (2013a). The ape that thought it was a peacock: does evolutionary psychology exaggerate human sex differences? Psychol. Inq. 24, 137–168. doi: 10.1080/1047840X.2013.804899

[ref336] Stewart-Williams S. Thomas A. G. (2013b). The ape that kicked the hornet's nest: response to commentaries on “the ape that thought it was a peacock”. Psychol. Inq. 24, 248–271. doi: 10.1080/1047840X.2013.823831

[ref337] Sugimoto T. Kobayashi H. Nobuyoshi N. Kiriyama Y. Takeshita H. Nakamura T.. (2009). Preference for consonant music over dissonant music by an infant chimpanzee. Primates 51, 7–12. doi: 10.1007/s10329-009-0160-3 19626392

[ref338] Suraci J. P. Clinchy M. Zanette L. Y. Wilmers C. C. (2019). Fear of humans as apex predators has landscape-scale impacts from mountain lions to mice. Ecol. Lett. 22, 1578–1586. doi: 10.1111/ele.13344, PMID: 31313436

[ref339] Tagg N. McCarthy M. Dieguez P. Agbor A. (2018). Nocturnal activity in wild chimpanzees (Pan troglodytes): evidence for flexible sleeping patterns and insights into human evolution. Am. J. Phys. Anthropol. 166, 510–529. doi: 10.1002/ajpa.23478, PMID: 29989158

[ref340] Tan Y. T. McPherson G. E. Peretz I. Berkovic S. F. Wilson S. J. (2014). The genetic basis of music ability. Front. Psychol. 5:658. doi: 10.3389/fpsyg.2014.00658, PMID: 25018744 PMC4073543

[ref341] Tobias J. A. Gamarra-Toledo V. García-Olaechea D. Pulgarin P. C. Seddon N. (2011). Year-round resource defence and the evolution of male and female song in suboscine birds: social armaments are mutual ornaments. J. Evol. Biol. 24, 2118–2138. doi: 10.1111/j.1420-9101.2011.02345.x, PMID: 21707816

[ref342] Tobias J. A. Montgomerie R. Lyon B. E. (2012). The evolution of female ornaments and weaponry: social selection, sexual selection and ecological competition. Philos. Trans. R. Soc. B: Biol. Sci. 367, 2274–2293. doi: 10.1098/rstb.2011.0280 PMC3391421 22777016

[ref343] Tonna M. Ponzi D. Palanza P. Marchesi C. Parmigiani S. (2020). Proximate and ultimate causes of ritual behaviour. Behav. Brain Res. 393:112772. doi: 10.1016/j.bbr.2020.112772, PMID: 32544508

[ref344] Tooby J. (2020). Evolutionary psychology as the crystalizing core of a unified modern social science. Evol. Behav. Sci. 14, 390–403. doi: 10.1037/ebs0000250

[ref345] Tooby J. Cosmides L. (2008). “The evolutionary psychology of the emotions and their relationship to internal regulatory variables” in Handbook of emotions. eds. Lewis M. Haviland-Jones J. M. Barrett L. F. (New York: Guilford Press), 114–137.

[ref346] Toups M. A. Kitchen A. Light J. E. Reed D. L. (2011). Origin of clothing lice indicates early clothing use by anatomically modern humans in Africa. Mol. Biol. Evol. 28, 29–32. doi: 10.1093/molbev/msq234 20823373 PMC3002236

[ref347] Trainor L. J. (2015). The origins of music in auditory scene analysis and the roles of evolution and culture in musical creation. Philos. Trans. R. Soc. B 370:0089. doi: 10.1098/rstb.2014.0089 PMC4321130 25646512

[ref348] Trainor L. J. (2021). Understanding the origins of musicality requires reconstructing the interactive dance between music-specific adaptations, exaptations, and cultural creations. Behav. Brain Sci. 44:E116. doi: 10.1017/S0140525X20001363 34588065

[ref349] Trehub S. E. (2003). The developmental origins of musicality. Nat. Neurosci. 6, 669–673. doi: 10.1038/nn1084, PMID: 12830157

[ref350] Treves A. Palmqvist P. (2007). “Reconstructing hominin interactions with mammalian carnivores (6.0–1.8 Ma)” in Primate Anti-Predator Strategies. eds. Gurski S. Nekaris K. A. I. (New York: Springer), 355–381.

[ref351] Tucker A. M. Feuerstein R. Mende-Siedlecki P. Ochsner K. N. Stern Y. (2012). Double dissociation: circadian off-peak times increase emotional reactivity; aging impairs emotion regulation via reappraisal. Emotion 12, 869–874. doi: 10.1037/a0028207 22642354 PMC3758763

[ref352] Twomey T. (2013). The cognitive implications of controlled fire use by early humans. Camb. Archaeol. J. 23, 113–128. doi: 10.1017/S0959774313000085

[ref353] Valentova J. V. Mafra A. L. Varella M. A. C. (2021). Enhancing the evolutionary science of self-presentation modification. Arch. Sex. Behav. 51, 79–84. doi: 10.1007/s10508-021-01975-0 33738591

[ref354] Valentova J. V. Tureček P. Varella M. A. C. Šebesta P. Mendes F. D. C. Pereira K. J.. (2019). Vocal parameters of speech and singing covary and are related to vocal attractiveness, body measures, and sociosexuality: a cross-cultural study. Front. Psychol. 10:2029. doi: 10.3389/fpsyg.2019.02029 31695631 PMC6817625

[ref9009] Van Bohemen S. Den Hertog L. van Zoonen L. (2018). Music as a resource for the sexual self: an exploration of how young people in the Netherlands use music for good sex. Poetics, 66, 19–29. doi: 10.1016/j.poetic.2017.12.001

[ref355] Vanderhoff E. N. Bernal H. N. (2022). Perspectives on antiphonal calling, duetting and counter-singing in non-primate mammals: an overview with notes on the coordinated vocalizations of bamboo rats (Dactylomys spp., Rodentia: Echimyidae). Front. Ecol. Evol. 10:906546. doi: 10.3389/fevo.2022.906546

[ref356] Varella M. A. C. (2018). The biology and evolution of the three psychological tendencies to anthropomorphize biology and evolution. Front. Psychol. 9:1839. doi: 10.3389/fpsyg.2018.01839 30327628 PMC6174228

[ref357] Varella M. A. C. (2021). Evolved features of artistic motivation: analyzing a Brazilian database spanning three decades. Front. Psychol. 12:769915. doi: 10.3389/fpsyg.2021.769915, PMID: 34992565 PMC8724029

[ref358] Varella M. A. C. (2022). Artistic motivations are intrinsic, specific, and temporally stable by nature: evidence from large real-life Brazilian public data between 1987–2004. Cult. Evol. 19, 68–80. doi: 10.1556/2055.2022.00012

[ref359] Varella M. A. C. Ferreira J. H. B. P. Cosentino L. A. M. Ottoni E. Bussab V. S. R. (2010). Sex differences in aspects of musicality in a Brazilian sample: adaptative hypotheses. Cogn. Music. Arts 4, 10–16.

[ref360] Varella M. A. C. Ferreira J. H. B. P. Souza A. A. L. (2014). The role of male and female in reproduction, and understanding of sexual selection when applied to human artistic propensities. Rock Art Res. 31, 239–240.

[ref361] Varella M. A. C. Luoto S. Soares R. B. S. Valentova J. V. (2021). COVID-19 pandemic on fire: evolved propensities for nocturnal activities as a liability against epidemiological control. Front. Psychol. 12:646711. doi: 10.3389/fpsyg.2021.646711, PMID: 33828510 PMC8019933

[ref362] Varella M. A. C. Santos I. B. C. Ferreira J. H. B. P. Bussab V. S. R. (2013). Misunderstandings in applying evolution to human mind and behavior and its causes: a systematic review. EvoS J. 5, 81–107.

[ref363] Varella M. A. C. Souza A. A. L. Ferreira J. H. B. P. (2011). Evolutionary aesthetics and sexual selection in the evolution of rock art aesthetics. Rock Art Res. 28, 153–163.

[ref364] Varella M. A. C. Souza A. A. L. Ferreira J. H. B. P. (2012). Considering both proximal and distal explanations for (rock) art production and appreciation as fruitful. Rock Art Res. 29, 227–229.

[ref365] Varella M. A. C. Štěrbová Z. Bártová K. Fisher M. Valentova J. V. (2022). Evolution of artistic and athletic propensities: testing of intersexual selection and intrasexual competition. Front. Psychol. 13:925862. doi: 10.3389/fpsyg.2022.925862, PMID: 35874330 PMC9301230

[ref366] Varella M. A. C. Valentova J. V. (2023). “Because the night belongs to sex, ‘drugs’ and rock ‘n’ roll: the nocturnal integration of promiscuity, psychoactive substance-seeking and musicality in human evolution” in Musical psychedelia research at the intersection of music and psychedelic experience. Ed. Farrell G. L. (London: Taylor and Francis Press), 192–210.

[ref367] Varella M. A. C. Valentova J. V. Fernández A. M. (2017). “Evolution of artistic and aesthetic propensities through female competitive ornamentation” in The Oxford handbook of female competition. ed. Fisher M. L. (New York, NY: Oxford University Press), 757–783.

[ref368] Verpooten J. (2021). Complex vocal learning and three-dimensional mating environments. Biol. Philos. 36, 78–86. doi: 10.1007/s10539-021-09786-2

[ref369] Verpooten J. Eens M. (2021). Singing is not associated with social complexity across species. Behav. Brain Sci. 44:E92. doi: 10.1017/S0140525X20001120 34588055

[ref370] Videan E. N. Fritz J. Howell S. Murphy J. (2007). Effects of two types and two genre of music on social behavior in captive chimpanzees (*Pan troglodytes*). J. Am. Assoc. Lab. Anim. Sci. 46, 66–70. PMID: 17203919

[ref371] Vink J. M. Groot A. S. Kerkhof G. A. Boomsma D. I. (2001). Genetic analysis of morningness and eveningness. Chronobiol. Int. 18, 809–822. doi: 10.1081/cbi-100107516 11763988

[ref372] Wade T. J. Fisher M. L. Salmon C. Downs C. (2021). Want to hookup?: sex differences in short-term mate attraction tactics. Evol. Psychol. Sci. 7, 430–438. doi: 10.1007/s40806-021-00282-0

[ref373] Wade T. J. Weinstein E. Dalal N. Salerno K. J. (2015). I can dance: further investigations of the effect of dancing ability on mate value. Hum. Ethol. Bull. 30, 10–20.

[ref374] Waller S. J. (2002). Psychoacoustic influences of the echoing environments of prehistoric art. J. Acoust. Soc. Am. 112:2284. doi: 10.1121/1.4779166

[ref375] Wallin N. L. (1991). Biomusicology. Neurophysiological, neuropsychological, and evolutionary perspectives on the origins and purposes of music. Stuyvesant, NY: Pendragon Press.

[ref376] Wallin N. L. Merker B. Brown S. (2000). The origins of music. Cambridge, MA: MIT Press.

[ref377] Wan C. Lalumière M. L. (2017). Can music cue sexual arousal? Can. J. Hum. Sex. 26, 238–248. doi: 10.3138/cjhs.2017-0011

[ref378] Wang S. C. Chern J. Y. (2008). The ‘night-owl’ learning style of art students: creativity and daily rhythm. Int. J. Art Des. Educ. 27, 202–209. doi: 10.1111/j.1476-8070.2008.00575.x

[ref379] Wang C. K. J. Tan L. Dairianathan E. I. (2018). Achievement goals, implicit theories, and intrinsic motivation: a test of domain specificity across music, visual art, and sports. J. Res. Music. Educ. 66, 320–337. doi: 10.1177/0022429418784563

[ref380] WebsterG. D.GraberJ. A.GesselmanA. N.CrosierB. S.SchemberT. O. (2014). A life history theory of father absence and menarche: a meta-analysis. Evol. Psychol. 12:147470491401200202. doi: 10.1177/14747049140120020225299880

[ref381] Weege B. Lange B. P. Fink B. (2012). Women’s visual attention to variation in men’s dance quality. Pers. Individ. Differ. 53, 236–240. doi: 10.1016/j.paid.2012.03.011

[ref382] Werner G. M. Todd P. M. (1997). “Too many love songs: sexual selection and the evolution of communication,” in Fourth European Conference on Artificial Life (Cambridge, MA: MIT Press, Bradford Books), 434–443.

[ref383] Wesseldijk L. W. Abdellaoui A. Gordon R. L. 23andMe Research Team Aslibekyan S. Auton A.. (2022). Using a polygenic score in a family design to understand genetic influences on musicality. Sci. Rep. 12:14658. doi: 10.1038/s41598-022-18703-w, PMID: 36038631 PMC9424203

[ref384] West-Eberhard M. J. (2014). Darwin's forgotten idea: the social essence of sexual selection. Neurosci. Biobehav. Rev. 46, 501–508. doi: 10.1016/j.neubiorev.2014.06.015, PMID: 25003806

[ref385] Wiessner P. W. (2014). Embers of society: firelight talk among the Ju/'hoansi bushmen. Proc. Natl. Acad. Sci. U. S. A. 111, 14027–14035. doi: 10.1073/pnas.1404212111, PMID: 25246574 PMC4191796

[ref386] Winegard B. Winegard B. Geary D. C. (2018). The status competition model of cultural production. Evol. Psychol. Sci. 4, 351–371. doi: 10.1007/s40806-018-0147-7, PMID: 37370044

[ref387] Wrangham R. (2009). Catching fire: How cooking made us human. Oxford: Basic Books.

[ref388] Wrangham R. (2017). Control of fire in the paleolithic: evaluating the cooking hypothesis. Curr. Anthropol. 58, S303–S313. doi: 10.1086/692113

[ref389] Wright S. E. Palmer C. (2022). Does chronotype explain daily timing of music behaviors? Chronobiol. Int. 39, 186–197. doi: 10.1080/07420528.2021.1989449, PMID: 34674591

[ref390] Wu Y. Wang H. Wang H. Hadly E. A. (2017). Rethinking the origin of primates by reconstructing their diel activity patterns using genetics and morphology. Sci. Rep. 7, 1–12. doi: 10.1038/s41598-017-12090-3 28928374 PMC5605515

[ref391] Xygalatas D. (2015). The biosocial basis of collective effervescence: an experimental anthropological study of a fire-walking ritual. Fieldw. Rel. 9, 53–67. doi: 10.1558/fiel.v9i1.53

[ref392] Yan R. Jessani G. Spelke E. S. de Villiers P. de Villiers J. Mehr S. A. (2021). Across demographics and recent history, most parents sing to their infants and toddlers daily. Philos. Trans. R. Soc. B 376:20210089. doi: 10.1098/rstb.2021.0089 PMC8558774 34719251

[ref393] Yoshida S. Okanoya K. (2005). Evolution of turn-taking: a bio-cognitive perspective. Cogn. Stud. 12, 153–165. doi: 10.11225/jcss.12.153

[ref394] Zahavi A. Zahavi A. (1999). The handicap principle: A missing piece of Darwin's puzzle. New York, NY, Oxford: Oxford University Press.

[ref395] Zamma K. (2014). What makes wild chimpanzees wake up at night? Primates 55, 51–57. doi: 10.1007/s10329-013-0367-1, PMID: 23817693

[ref396] Zatorre R. J. (2015). Musical pleasure and reward: mechanisms and dysfunction. Ann. N. Y. Acad. Sci. 1337, 202–211. doi: 10.1111/nyas.12677, PMID: 25773636

[ref397] Zhong C. B. Bohns V. K. Gino F. (2010). Good lamps are the best police: darkness increases dishonesty and self-interested behavior. Psychol. Sci. 21, 311–314. doi: 10.1177/0956797609360754, PMID: 20424061

[ref398] Zohar I. Alperson-Afil N. Goren-Inbar N. Prévost M. Tütken T. Sisma-Ventura G.. (2022). Evidence for the cooking of fish 780,000 years ago at Gesher Benot Ya’aqov. Israel. Nat. Ecol. Evol. 6, 2016–2028. doi: 10.1038/s41559-022-01910-z, PMID: 36376603

